# Three-Level Fish Iris Image Annotatability Assessment for Pre-Annotation Quality Control Based on Boundary Traceability

**DOI:** 10.3390/s26196053

**Published:** 2026-09-24

**Authors:** Ziying Yang, Yuyang Wu, Ying Liang

**Affiliations:** School of Aerospace Science and Engineering, Shandong University, Weihai 264209, China; 202300800188@mail.sdu.edu.cn (Z.Y.); 202300800319@mail.sdu.edu.cn (Y.W.)

**Keywords:** underwater fish iris imaging, image annotatability assessment, boundary traceability, pre-annotation quality control, no-reference image quality assessment, quality evidence integration

## Abstract

**Highlights:**

**Why are conventional no-reference image quality scores insufficient for fish iris annotatability assessment?**

- They provide global perceptual quality evaluation but cannot characterize boundary traceability required for annotation readiness.

**How can heterogeneous quality evidence be integrated under limited data conditions?**

- Independent learning of handcrafted and deep quality evidence with parameter-free posterior averaging provides an effective solution.

**What is the role of boundary-traceability-based pre-annotation screening?**

- It supports efficient labeling-resource allocation for fine-grained biological dataset construction.

**Why is decision-level posterior averaging adopted?**

- It enables simple and robust integration of heterogeneous evidence when task-specific labels are limited.

**Abstract:**

Fish iris images provide morphological information for biometric recognition and biological observation, but underwater imaging often introduces blur, reflection, occlusion, low contrast, and boundary ambiguity. Conventional no-reference image quality assessment provides global perceptual scores but cannot determine whether local iris boundaries are reliably traceable for annotation. We formulate fish iris quality control as a three-level annotatability assessment task for pre-annotation screening, enabling direct annotation, further review, or exclusion/reacquisition. Under limited supervision, the framework learns complementary quality evidence from 53-dimensional handcrafted quality descriptors and 384-dimensional embeddings extracted from a frozen pretrained MANIQA (Multi-Dimension Attention Network for No-Reference Image Quality Assessment) model. LateAvg performs lightweight decision-level integration by equally averaging branch posterior probabilities. On 1500 self-collected underwater images evaluated by fish-group five-fold cross-validation, the framework achieves 0.8574 accuracy and 0.8510 Macro-F1. It achieves the highest mean Accuracy, Macro-F1, and Weighted-F1 among evaluated methods, outperforming scalar IQA baselines such as NIQE-score and TOPIQ-score while remaining statistically comparable to trainable integration and late fine-tuning approaches. These results support boundary-traceability-based pre-annotation screening. With cached quality evidence, the framework introduces only a lightweight decision stage to support efficient annotation-resource allocation in biological dataset construction.

## 1. Introduction

Fish iris images provide fine-grained morphological information for individual identification and biological observation [1,2]. Beyond biometric applications, variations in ocular morphology and appearance may provide visual cues associated with physiological or behavioral variations in fish, although establishing such biological associations requires dedicated validation beyond the scope of this study. The present study does not claim that iris appearance alone provides a validated indicator of fish health or stress; establishing such biological associations would require dedicated physiological validation beyond the scope of this work.

No-reference image quality assessment (NR-IQA) provides a natural starting point, with handcrafted statistics and deep representations both targeting global perceptual degradation [3,4]. However, perceptual quality and annotation readiness are fundamentally different objectives. An image with acceptable overall clarity can still be difficult to label if a critical segment of the iris boundary is obscured by reflection or confused with surrounding tissue; conversely, moderate global degradation does not prevent reliable delineation when both boundaries remain distinguishable. Similar structure-dependent quality requirements have been reported in other biomedical imaging tasks [5,6]. For fish iris annotation, the relevant question is not how good the image looks, but whether the inner and outer boundaries are simultaneously traceable—a question that general IQA scores are not designed to answer.

Existing data-quality work addresses three largely post-annotation concerns: crowdsourcing agreement and annotator modeling [7,8,9], label-error detection and noisy-label learning [10,11,12,13], and dataset verification after initial labeling [14,15]. Biometric sample utility, by comparison, is typically defined by downstream recognition accuracy [16], and interactive annotation tools reduce effort only once a sample has been accepted for labeling [17]. None of these lines of work provide a structured pre-annotation admission decision—accept, review, or reject—based on target-boundary evidence before manual annotation starts. This pre-annotation screening gap is particularly acute for fine-grained biological imaging, where annotation is expensive, and boundary ambiguity is widespread.

We formulate fish iris annotatability as a three-level pre-annotation screening task—High for direct annotation, Medium for further review, Low for exclusion or reacquisition—aligned with practical dataset-construction workflows. To cope with limited task-specific labels, we develop a quality-evidence integration framework that learns complementary information from 53-dimensional handcrafted quality descriptors (HQD) and 384-dimensional frozen deep quality embeddings (DQE) extracted from pretrained MANIQA [18]. A lightweight decision-level integration strategy, termed LateAvg, is adopted to combine the two evidence branches by equally averaging their class posterior probabilities after branch-wise probability normalization. We evaluate the approach on 1500 self-collected underwater fish images using fish-group five-fold cross-validation to prevent identity-level leakage. The results show that integrating complementary quality evidence provides an effective and parsimonious solution for limited-data annotatability assessment, substantially outperforming scalar IQA baselines while remaining competitive with trainable feature-integration approaches.

The main contributions of this work are threefold:

1. Task formulation. We define fish iris image annotatability as a three-level, workflow-aligned screening problem based on inner- and outer-boundary traceability, shifting pre-annotation quality control from global perceptual scoring to target-structure usability assessment.
2. Dataset and evaluation protocol. We construct a 1500-image task-specific dataset with auxiliary geometric reliability annotations and adopt fish-group five-fold cross-validation to assess generalization to unseen fish individuals, providing a controlled evaluation setting for pre-annotation screening research.
3. Validation and practical analysis. Through systematic comparisons with feature-level integration and late fine-tuning approaches, we demonstrate the effectiveness of integrating heterogeneous quality evidence for fish iris annotatability assessment. Additional reliability and efficiency analyses further validate the feasibility of the proposed framework for practical pre-annotation quality control.

## 2. Related Work

Research related to fish iris image annotatability mainly involves three directions: no-reference image quality assessment and quality representation, annotation quality and image utility, and heterogeneous evidence learning under limited data.

### 2.1. No-Reference IQA and Quality Representations

Classical no-reference image quality assessment (NR-IQA) methods mainly rely on natural-scene statistics, frequency-domain regularities, or handcrafted perceptual descriptors. Representative methods include BIQI, DIIVINE, BLIINDS-II, BRISQUE, NIQE, ILNIQE, and PIQE [3,19,20,21,22,23,24]. Underwater-specific metrics, such as UCIQE and UIQM, further characterize color attenuation, contrast loss, and sharpness degradation in aquatic environments [25,26]. These methods are generally lightweight and interpretable, but primarily reduce image quality to a global scalar value. Recent reviews similarly indicate that underwater IQA remains largely centered on perceptual quality or enhancement evaluation [27].

Deep NR-IQA has evolved from patch-based CNNs and end-to-end quality prediction [28,29,30,31,32] toward ranking-based, bilinear, adaptive, and uncertainty-aware modeling [33,34,35,36,37]. Recent methods further employ Transformer reasoning, visual compensation, distortion-aware convolution, and supervised contrastive learning [4,38,39,40,41]. Models such as Transformer for IQA, MUSIQ, MANIQA, MSTRIQ, and TOPIQ capture multi-scale or semantic quality information through attention-based representations [18,42,43,44]. Beyond final scores, intermediate embeddings can retain richer spatial and distortion-aware information; however, both scalar scores and generic deep embeddings are learned for perceptual quality objectives rather than for determining whether task-specific structures in biological images, such as inner and outer fish iris boundaries, are simultaneously distinguishable and traceable. Task-specific adaptation is therefore required for annotatability assessment.

### 2.2. Annotation Quality, Image Utility, and Pre-Annotation Screening

Research on annotation quality spans three complementary directions: annotator modeling, label-error correction, and annotation assistance. These studies are particularly relevant to biological image analysis, where manual annotation is often expensive and domain expertise is required; however, most existing approaches focus on improving or evaluating annotations after labeling has begun rather than assessing whether an image is suitable for annotation beforehand.

Annotator modeling and crowdsourcing. When multiple annotators are available, inter-annotator disagreement can be modeled explicitly to estimate individual reliability and aggregate inconsistent labels [7,8,9]. These methods are valuable for crowdsourcing pipelines but assume annotation has already been initiated and multiple labelers are available—conditions that do not hold in the present single-expert, pre-annotation setting.

Label-error detection and noisy-label learning. A second line of work identifies mislabeled samples or learns robustly in the presence of label noise after a dataset has been constructed [10,11,12,13]. Dataset verification studies further examine how annotation quality affects downstream model performance [14,15]. These post-construction analyses are complementary to our goal of screening samples before they enter the labeling pipeline.

Image utility and annotation assistance. Biometric sample quality is typically defined by its impact on automatic recognition performance [16], which differs from annotation readiness because a recognizable sample may still be difficult to delineate. Interactive segmentation and annotation tools, meanwhile, reduce labeling cost but operate only after a sample has been accepted for annotation [17].

Distinction from the present work. All of the above approaches address problems that arise during or after annotation. By contrast, this study targets the earlier admission decision: whether an image contains sufficiently traceable iris boundaries to justify manual delineation in the first place. The three-level High/Medium/Low scheme therefore provides a pre-annotation assessment mechanism that complements existing post-annotation quality control and annotation-efficiency optimization approaches.

### 2.3. Heterogeneous Evidence Learning Under Limited Data

Combining handcrafted and deep representations has been explored in several IQA and visual recognition contexts, motivated by the complementary strengths of explicit statistics and implicit learned features. Handcrafted descriptors provide interpretable task-specific cues, whereas pretrained embeddings capture richer quality patterns but may require adaptation for specialized tasks. In the present setting, frozen MANIQA representations preserve higher-level quality information without full backbone adaptation [18], while HQD explicitly characterizes local sharpness, contrast, edge response, and texture properties tied to iris-boundary traceability.

Several related heterogeneous IQA methods exist. STNS-IQA, for example, combines natural-scene statistics with Swin-Transformer features for no-reference perceptual quality prediction [45]. It differs from our framework in three respects: it targets scalar quality regression rather than three-level annotatability classification; its statistical features describe general natural-image regularities rather than boundary traceability; and its heterogeneous features are integrated for perceptual quality prediction rather than for workflow-oriented annotatability assessment. Other multi-branch IQA architectures similarly explore feature-level or decision-level integration strategies [33,36], whereas we deliberately preserve separate branch predictors and combine their posterior probabilities only at the decision stage. Broadly, multimodal and multi-representation fusion can be categorized as feature-level, representation-level, and decision-level [46]. Feature concatenation and shared or gated latent representations enable direct interaction between evidence sources but introduce additional trainable parameters and require heterogeneous features to be aligned in a common space. Decision-level fusion, by contrast, preserves branch-specific predictors and combines their outputs after each representation has formed an independent class estimate. This distinction is important under limited supervision and heterogeneous evidence distributions.

Rather than forcing HQD and DQE into a shared latent space, we retain independent predictors and combine their class posteriors through parameter-free equal averaging. Decision-level posterior averaging is not proposed as a new fusion algorithm nor claimed to be universally optimal. Instead, we empirically evaluate whether this simplest decision-level strategy suffices for limited-data fish iris annotatability assessment, benchmarking it against single-branch, feature-level gated fusion, and partial-fine-tuning alternatives.

Taken together, the existing literature leaves two gaps that the present work addresses. First, existing studies have rarely considered fish iris image quality from the perspective of boundary-traceable annotatability with explicit workflow-aligned grading. The following sections describe the dataset, task formulation, and LateAvg framework designed to fill these gaps.

## 3. Dataset and Task Definition

### 3.1. Fish Iris Image Acquisition and Dataset Characteristics

This study addresses fish iris image annotatability assessment as a data-centric pre-annotation quality-control task. The objective is to determine whether the fish eye/iris region of an acquired image provides sufficient visual evidence for reliable manual delineation before the image enters the labeling stage. Unlike conventional perceptual image quality assessment, the proposed task focuses specifically on whether the inner and outer iris boundaries can be consistently traced by human annotators.

The task-specific evaluation dataset was constructed from self-collected underwater fish images acquired using a custom-built non-contact fish iris imaging system. The configuration of the acquisition system is illustrated in Figure 1. It covers two fish species: neon dwarf rainbowfish (*Melanotaenia praecox*) and zebrafish (*Danio rerio*). Detailed specifications of the acquisition system are provided in Supplementary Table S1. Although the original images contain the full fish body, annotatability is assessed according to the local eye/iris region. A total of 1500 images were selected from the self-collected image pool, comprising 500 samples for each annotatability level: High, Medium, and Low. Public fish-eye datasets specifically designed for annotatability assessment, including explicit iris-boundary annotation and annotatability-level labels, are currently unavailable. Therefore, we constructed a task-specific dataset to investigate this newly formulated pre-annotation quality control problem. Rather than serving as a replacement for existing fish recognition benchmarks, the dataset is designed to provide controlled evaluation of boundary-traceability-based annotation screening.

Although the acquisition system was designed to provide relatively stable underwater imaging conditions, images captured during near-natural fish movement exhibit diverse variations that affect annotatability. Typical factors include motion blur, specular reflection, uneven illumination, local shadow, partial occlusion, low contrast, ambiguous iris boundaries, incomplete eye-region visibility, and missing or undetectable iris structures. These variations directly affect whether the inner and outer iris boundaries can be reliably delineated. The resulting dataset therefore serves as a task-specific evaluation dataset for fish iris annotatability assessment rather than as a conventional perceptual image quality dataset.

### 3.2. Fish Iris Image Annotatability Definition

Fish iris image annotatability is defined as the degree to which an image provides sufficient delineation evidence of the fish iris region. In this study, the annotation target is the iris area represented by the annular region between the inner and outer iris boundaries. Annotatability is therefore different from conventional perceptual image quality, which measures overall visual appearance, and from image utility assessment, which is usually defined by downstream model performance.

Given an input underwater fish image Ii, annotatability assessment is formulated as a three-class image-level classification problem:

(1) $$y_{i}\in C,C=\{H,M,L\},$$

where H, M, and L denote High, Medium, and Low annotatability, respectively. Although the input image contains the full fish body, its annotatability label is determined by the visibility, clarity, and boundary distinguishability of the local eye/iris region. Each image is therefore assigned to one of three operational outcomes: suitability for direct manual annotation, the need for further human review, or exclusion or reacquisition. Table 1 summarizes the definitions and visual criteria of the three annotatability levels.

Representative examples of the three annotatability levels are shown in Figure 2. The examples illustrate that full-body fish images are categorized according to the visibility and boundary traceability of the local eye/iris region rather than the overall visibility of the fish.

### 3.3. Annotation Protocol and Reliability-Aware Label Construction

Stage 1: Image-level annotatability labeling.

The image-level annotatability label was assigned by a senior researcher. Before formal labeling, the annotator completed a calibration session on a 50-image pilot set using the criteria in Table 1, with edge cases reviewed iteratively until the grading standard stabilized. Each image was then classified as High, Medium, or Low based on the visibility and traceability of the inner and outer iris boundaries. Because H/M/L grading involves inherent ordinal subjectivity—especially at the Medium–Low boundary—these labels should be interpreted as a consistent operational standard from one domain expert rather than a multi-assessor gold standard. The resulting label was stored as manual_label and used as the fixed hard ground-truth target for all evaluation procedures. The H/M/L labels were assigned independently of the geometric iris annotations, the Annotation Reliability Score, and the YOLO detection outputs, and remained fixed throughout all training, validation, and evaluation procedures.

The image-level annotatability label and geometric iris annotation serve different purposes in this study. The former defines the primary classification target, representing whether an image is suitable for annotation under the operational High/Medium/Low criteria. The latter provides auxiliary structural evidence for quantifying annotation consistency and constructing reliability-aware supervision. Therefore, geometric annotations were not used to redefine the H/M/L labels, but only to characterize boundary delineation reliability and support reliability-aware model training.

Stage 2: Geometric iris boundary annotation.

To obtain auxiliary structural reliability information, three additional annotators independently attempted to delineate the fish iris region in each image using LabelMe. Each geometric annotation consisted of an inner circular boundary and an outer circular boundary, with the annular area between them treated as the iris region. Depending on whether a valid iris structure could be identified, each image contained up to three valid geometric annotations. These annotations were not used to vote on or redefine the H/M/L annotatability label. Instead, they were used to quantify the consistency of iris-region localization and boundary delineation across annotators and to construct auxiliary reliability information for model training.

To measure geometric annotation reliability, an Annotation Reliability Score (ARS) was calculated for each image. The annular iris regions annotated by different annotators were first converted into binary masks, and the mean pairwise intersection-over-union between valid annular masks was computed as ${\bar{IoU}}_{i}$. The geometric stability of the annotated iris structures was further characterized using normalized variations of the circle centers and radii. Considering both annotation completeness and geometric consistency, the ARS for the i-th image was defined as:

(2) $${ARS}_{i}=\frac{n_{i}}{3}\cdot IoU_{i}\cdot exp(-\sigma_{c,i}^{norm}-\;\sigma_{r,i}^{norm}),$$

where $n_{i}$ denotes the number of valid geometric annotations for image $I_{i}$, $IoU_{i}$ represents the mean pairwise intersection-over-union, and $\sigma_{c,i}^{norm}$ and $\sigma_{r,i}^{norm}$ denote the normalized variations of circle centers and radii, respectively. A higher ARS indicates stronger inter-annotator consistency in iris-region localization and boundary delineation. For images without a valid geometric annotation, $n_{i}=0$ and $ARS_{i}$ are set to zero. In this study, ARS is used as an indicator of geometric annotation consistency for reliability-aware soft-target construction and sample weighting, rather than as a direct measure of annotation time, annotation cost, or downstream model performance. The exponential form is chosen so that geometric instability penalizes the score multiplicatively rather than additively, preventing a single unstable annotation from being compensated by a high IoU on the remaining pair. The normalization of $\sigma_{c}$ and $\sigma_{r}$ to [0, 1] is performed fold-wise using training-split statistics, consistent with the fold-safe protocol described below.

For model training, the raw ARS values were converted into normalized reliability scores ${ARS}_{i}^{norm}\in \left[0,1\right]$. To prevent information leakage, the normalization mapping was estimated independently within each cross-validation fold using only the corresponding training split—a procedure hereafter referred to as fold-safe normalization—and was then applied unchanged to the associated validation and test splits. The manually assigned hard label, stored as manual_label, remained unchanged for all 1500 images. Table 2 summarizes the number of valid geometric annotations and the reliability-score statistics in the checked dataset records.

For reliability-aware neural-model training, the fold-safe normalized reliability score ${ARS}_{i}^{norm}$, together with the ROI-localization uncertainty cue, was used to construct the soft-target distribution and sample weight. This procedure did not alter the ground-truth class of any image; instead, it adjusted the strength and certainty of the training supervision according to geometric annotation consistency and ROI-localization reliability. In the final checked dataset records, the dominant class of every corrected soft target remained consistent with manual_label.

The reliability-aware soft targets and sample weights were used only during the training of neural single-branch predictors and trainable fusion references. They were not used as evaluation targets and did not directly participate in decision-level posterior averaging. All reported performance metrics were computed strictly against the fixed hard label manual_label. This design allows the neural models to use auxiliary annotation-reliability information during training while preserving a fixed and interpretable H/M/L evaluation target.

### 3.4. Dataset Organization and Auxiliary Information

Because the original images contain the full fish body and irrelevant background, annotatability-related quality evidence was extracted from a localized eye region of interest (ROI) whenever available. The auxiliary metadata and cached quality-evidence fields associated with each sample are summarized in Supplementary Table S2. The ROIs were generated offline using a fixed YOLOv8n detector trained on a separately prepared eye-detection dataset with no image-level or fish-identity-level overlap with the 1500-image main-task dataset. The configuration of the fixed eye-region localization module is provided in Supplementary Table S3. The detector was not jointly optimized with any annotatability assessment model, and the same cached ROI/fallback records were used by all compared methods. The highest-confidence eye-region bounding box was accepted when its confidence was no lower than 0.25; otherwise, the complete original image was used as the fallback input. Valid ROIs were available for 1400 images (93.33%), while the remaining 100 images (6.67%) used the full-image fallback.

### 3.5. Fish-Group Five-Fold Cross-Validation Protocol

Standard image-level random cross-validation may introduce data leakage in this dataset because multiple images originate from the same fish individual and can share strong visual and acquisition-related similarities. To prevent identity-level overlap and obtain a more realistic estimate of generalization to unseen fish, a fish-group five-fold cross-validation protocol was adopted. Dataset partitioning was performed at the fish-identity level rather than at the individual-image level.

Specifically, the 40 fish individuals were partitioned into five disjoint test groups, while maintaining the species distribution across folds as evenly as possible. In each fold, one fish group was held out as the test set. The remaining fish identities were further assigned to the training and validation subsets to approximate an 80%/20% split of the non-test samples while preserving fish-level separation. Consequently, no fish individual appeared in more than one of the training, validation, or test subsets within the same fold. Identical fold assignments were used for all compared methods.

Table 3 summarizes the sample counts for each fold. Across the five mutually exclusive test folds, the total numbers of High, Medium, and Low samples are each 500, matching the balanced class distribution of the complete evaluation dataset. Compared with random image-level splitting, this protocol evaluates model performance on images from unseen fish individuals and reduces the risk of identity-related information leakage.

## 4. Proposed Method

### 4.1. Overview of the LateAvg Framework

Fish iris annotatability depends on both local visual cues and higher-level quality representations. Handcrafted Quality Descriptors (HQD) capture boundary-related characteristics such as intensity, illumination, sharpness, edge response, and texture, whereas Deep Quality Embeddings (DQE) retain quality-aware representations learned from a pretrained MANIQA model. The proposed LateAvg framework integrates 53-dimensional HQD and 384-dimensional DQE extracted from the frozen MANIQA backbone, preserving pretrained quality information while avoiding additional backbone optimization on the limited annotatability dataset. As illustrated in Figure 3, the framework consists of four main stages: eye-region selection, dual-source quality evidence extraction, independent branch classification, and decision-level posterior averaging. For each underwater fish image, an accepted eye-region ROI is used when available; otherwise, the complete image is retained as a fallback input. The selected region is then represented separately by HQD and DQE. Each representation is processed by its own classifier, producing an independent three-class logit vector over High, Medium, and Low annotatability. The posterior distribution of each branch is first obtained through independent softmax normalization. The final posterior distribution is then computed by equally averaging the two branch posterior probabilities, and the class with the highest averaged probability is selected as the final prediction. By preserving branch-specific representation learning and combining decisions only at the decision level, LateAvg avoids introducing additional trainable feature-fusion modules while retaining information from both explicit handcrafted descriptors and pretrained deep quality representations.

In this study, decision-level averaging is not regarded as a novel integration strategy. Instead, LateAvg uses equal-weight posterior averaging as a simple and parameter-free integration strategy to examine whether heterogeneous quality evidence can be effectively combined under limited task-specific supervision.

### 4.2. Implementation Details

To ensure reproducibility, the implementation details of the proposed LateAvg framework are provided. For deep quality evidence extraction, the selected eye-region ROI was used whenever available; otherwise, the full image was retained as the fallback input. The input region was resized to 224×224 pixels and normalized using the ImageNet mean and standard deviation before being fed into the frozen MANIQA backbone. Only the extracted 384-dimensional deep quality embedding was used for annotatability classification, while the MANIQA parameters remained unchanged.

The selected ROI/fallback image region was also used for HQD extraction. The resulting 53-dimensional descriptor was standardized independently within each cross-validation fold, where the feature-wise statistics were estimated only from the training split and applied unchanged to the validation and test splits. Features with near-zero training-set variance were assigned a standardized value of zero. All neural models were optimized using AdamW with a learning rate of $1\times 10^{-4}$, weight decay of $1\times 10^{-4}$, a batch size of 64, and a maximum of 50 epochs. Early stopping with a patience of 10 epochs and gradient clipping with a maximum norm of 1.0 were applied.

### 4.3. Dual-Source Quality Evidence Representation

As described in Section 3.4, each image is represented by an accepted eye-region ROI or, when unavailable, the complete image; the same selected region feeds both quality representations.

The first evidence source is a 53-dimensional Handcrafted Quality Descriptor (HQD), denoted by $q_{i}\in R^{53}$. It comprises six groups of task-oriented statistics, including gray-level intensity statistics, HSV-based illumination statistics, sharpness and edge statistics, noise-related statistics, GLCM (Gray-Level Co-Occurrence Matrix) texture features, and Gabor directional texture features, which characterize local intensity, illumination, sharpness, noise, texture, and directional structure. These properties are closely related to the visibility and traceability of the inner and outer iris boundaries. The descriptor composition is summarized in Table 4, while the definitions of all individual features are provided in Supplementary Table S4. The original HQD vector $q_{i}$ is standardized using fold-dependent training statistics, and the resulting standardized representation is denoted as $\bar{q_{i}}$.

To prevent information leakage, HQD standardization is performed independently within each cross-validation fold. The mean and standard deviation of each feature are estimated from the training split only and then applied unchanged to the corresponding validation and test splits. Features with near-zero training-set variance are assigned a standardized value of zero.

The second evidence source is a 384-dimensional Deep Quality Embedding (DQE), denoted by $d_{i}\in R^{384}$, extracted offline from a MANIQA model [18] pretrained on the KonIQ-10k database [47]. Rather than using the final scalar quality score, the token representation immediately preceding the quality-prediction heads is retained. The 784 spatial tokens are averaged to obtain a single 384-dimensional image-level vector. The MANIQA backbone remains frozen during feature extraction, and no annotatability labels are used to adapt its parameters.

HQD and DQE therefore provide explicit handcrafted evidence and implicit deep quality evidence, respectively. Because they originate from different representation spaces, they are retained as separate inputs to the subsequent branch classifiers rather than being directly concatenated or projected into a shared feature space.

### 4.4. Independent Branch Prediction and Decision-Level Posterior Averaging

The standardized HQD vector ${\bar{q}}_{i}$ and the DQE vector $d_{i}$ are processed by two independent branch-specific MLP predictors. The HQD branch maps the 53-dimensional handcrafted representation to a 128-dimensional hidden representation, whereas the DQE branch uses a separate projection to transform the 384-dimensional deep quality representation into the same hidden dimensionality. Each branch subsequently produces three logits corresponding to the annotatability class set C={H,M,L}. The branch logits are defined as:

(3) $$z_{i}^{HQD}=f_{HQD}\left(\tilde{q_{i}}\right),z_{i}^{DQE}=f_{DQE}\left(d_{i}\right),$$

where $f_{HQD}\left(\cdot \right)$ and $f_{DQE}\left(\cdot \right)$ denote the independent HQD and DQE branch classifiers, respectively, and $z_{i}^{HQD},z_{i}^{DQE}\in R^{3}$ represent the corresponding three-class logit vectors.

The posterior probability distribution of each branch is obtained by independently applying Softmax normalization to its logits:

(4) $$p_{i}^{HQD}=Softmax(z_{i}^{HQD}),{\;p}_{i}^{DQE}=Softmax(z_{i}^{DQE}).$$

The two predictors are optimized in separate training runs and do not share parameters, intermediate representations, or checkpoints. The HQD branch learns a task-specific decision mapping from explicit local quality statistics, whereas the DQE branch learns from the frozen MANIQA-based deep quality representation. Consequently, the two branches retain independent decision functions before their outputs are combined at the decision level.

At inference time, LateAvg equally averages the class posterior probabilities predicted by the two branches:

(5) $$p_{i}^{LateAvg}=\frac{1}{2}\left(p_{i}^{HQD}+p_{i}^{DQE}\right),$$

where $p_{i}^{LateAvg}\in {\left[0,1\right]}^{3}$ denotes the final class-posterior distribution obtained by equally averaging the posterior probabilities produced by the independently trained HQD and DQE branches. Because both $p_{i}^{HQD}$ and $p_{i}^{DQE}$ are normalized probability distributions, their equal-weight average is also a normalized probability distribution. Therefore, no additional Softmax normalization is applied after fusion.

The use of equal posterior averaging is motivated by the limited-data and heterogeneous-evidence setting of this study. Unlike trainable feature-level integration, this strategy introduces no additional fusion parameters and preserves the independently learned decision behavior of the two branches. HQD and DQE originate from substantially different representation spaces—explicit handcrafted quality descriptors and implicit pretrained deep quality embeddings, respectively—so decision-level integration avoids forcing these heterogeneous representations into an additional shared latent space. Accordingly, LateAvg is used here as a simple parameter-free integration strategy rather than as a novel fusion algorithm; its effectiveness relative to trainable fusion alternatives is evaluated empirically in Section 5.

The final annotatability prediction is obtained by selecting the class with the highest averaged posterior probability:

(6) $${\hat{y}}_{i}=argmax_{c\in C}p_{i,c}^{LateAvg},\;\;C=\{H,M,L\}.$$

Thus, LateAvg performs decision-level integration by equally averaging the posterior probabilities produced by two independently trained branches. It does not perform logit averaging, feature concatenation, shared representation learning, or additional trainable fusion, and no further probability normalization is required after posterior averaging.

### 4.5. Reliability-Aware Training of Neural Branch Predictors

For each sample i, the manually assigned annotatability label is denoted by $y_{i}\in C$, where $C=\left\{H,M,L\right\}$ represents the three annotatability classes. The hard label is used as the ground-truth target for model evaluation. During training, each sample is additionally associated with a reliability-aware soft target vector ${\tilde{\;y}}_{i}\in {\left[0,1\right]}^{\left|C\right|}$ and a scalar reliability weight $w_{i}$. For class c∈C, $y_{i,c}$ denotes the soft-target probability assigned to class c, satisfying

(7) $$\sum_{c\in C}{\tilde{y}}_{i,c}=1,$$

and the soft target does not change the corresponding manually assigned class label. The sample weight controls the contribution of each sample according to its annotation reliability. The construction of ${\tilde{y}}_{i}$ and $w_{i}$ is described in Supplementary Note S1. For each cross-validation fold, all fold-dependent reliability statistics are estimated from the training split only.

The HQD predictor is optimized using weighted soft-label cross-entropy. For a mini-batch M containing N samples, its objective is

(8) $$L_{HQD}=-\frac{\Sigma_{i\in M}w_{i}\Sigma_{c\in C}{\tilde{y}}_{i,c}logp_{i,c}^{HQD}}{\Sigma_{i\in M}w_{i}},$$

where i∈M indexes samples in the mini-batch and  c∈C denotes the annotatability class index; $p_{i,c}^{HQD}$, $w_{i}$, and $y_{i,c}$ represent the predicted probability, reliability weight, and soft-target probability, respectively.

The DQE predictor is trained independently using the same supervision rule:

(9) $$L_{DQE}=-\frac{\Sigma_{i\in M}w_{i}\Sigma_{c\in C}{\tilde{y}}_{i,c}logp_{i,c}^{DQE}}{\Sigma_{i\in M}w_{i}},$$

where $p_{i,c}^{DQE}\;$ denotes the DQE-branch predicted probability for class c.

The two branch objectives are optimized in separate training runs. No parameters, gradients, or checkpoints are shared between the HQD and DQE predictors, and no auxiliary branch loss or fusion-specific regularization is introduced. The reliability-aware targets and sample weights affect training only. During inference, LateAvg combines the independently predicted branch posterior probabilities through equal-weight posterior averaging.

## 5. Experiments

### 5.1. Experimental Setup

Experiments were conducted on the fish iris image annotatability dataset described in Section 3, comprising 1500 self-collected underwater images with three annotatability levels: High (H), Medium (M), and Low (L). All methods were evaluated using the same fish-group five-fold cross-validation partitions, where images from the same fish individual were restricted to a single training, validation, or test subset within each fold. Fold-dependent preprocessing statistics, including HQD feature standardization and reliability-score normalization, were estimated only from the training split and applied unchanged to the corresponding validation and test splits.

For conventional machine-learning baselines, RF, XGBoost, LR, and SVM were trained using standardized HQD features with fixed hard labels. Scalar-IQA baselines used the corresponding quality scores as inputs to three-class logistic regression classifiers. All classifier hyperparameters were selected using the training/validation splits within each fold, and no information from the held-out test split was used during model selection.

For neural models, the checkpoint with the highest validation Macro-F1 was selected for evaluation on the corresponding held-out fold. Accuracy, Macro-F1, Weighted-F1, and class-wise F1-scores were reported as mean ± standard deviation across the five folds. Macro-F1 was adopted as the primary metric because it assigns equal importance to the three annotatability classes. The detailed implementation settings of the proposed framework are summarized in Table 5. The detailed hyperparameter configurations of all competing methods are provided in Supplementary Table S7.

### 5.2. Baseline Evaluation and Main Comparison

This section compares scalar IQA scores, conventional HQD-based classifiers, neural single-branch models, fusion strategies, and image-level fine-tuning approaches under the same fish-group five-fold cross-validation protocol.

Table 6 shows that scalar IQA scores provide limited and strongly class-dependent evidence for annotatability assessment. NIQE-score achieves the highest Macro-F1 among these baselines, whereas TOPIQ-score performs best on Medium samples but remains weak on the Low class. The inconsistent class-wise behavior across traditional, underwater-specific, and deep IQA scores indicates that a single global quality value is insufficient to characterize the local visibility and boundary traceability required for fish iris annotation.

As Table 7 shows, the 53-dimensional HQD representation substantially outperforms the scalar IQA scores. LR-53D achieves the highest Macro-F1 among the conventional classifiers and is therefore retained as the representative HQD-based baseline in the main comparison. This result indicates that task-oriented multidimensional descriptors preserve annotatability information that is lost when image quality is reduced to a single scalar value.

Based on the preceding results, NIQE-score, TOPIQ-score, and LR-53D are retained as representative scalar-IQA and conventional HQD-based baselines. HQD-only and DQE-only represent the two neural evidence branches, while GMU-Fusion (Gated Multimodal Unit- Fusion) and MANIQA-LateStage-FT provide feature-level fusion and image-level fine-tuning references, respectively. The scalar-IQA and conventional machine-learning methods use their standard hard-label formulations, whereas the neural single-branch and fusion models share the same reliability-aware supervision. Cross-category comparisons therefore reflect the complete method configurations, while branch complementarity and fusion effectiveness are assessed primarily among neural models trained under the same supervision protocol.

Table 8 shows that task-oriented representations substantially outperform scalar IQA scores. NIQE-score reaches a Macro-F1 of 0.6139 ± 0.1003, whereas LR-53D and DQE-only achieve 0.8234 ± 0.0725 and 0.8385 ± 0.0516, respectively. Because these method categories use different supervision formulations, the cross-category gains should be interpreted as differences between complete assessment pipelines rather than as the effect of representation alone.

LateAvg obtains the highest mean Accuracy, Macro-F1, and Weighted-F1 among the compared methods, with a Macro-F1 of 0.8510 ± 0.0612; whether this ranking reflects a statistically reliable advantage over the strongest baselines is examined in Section 5.4. Under the same reliability-aware supervision, it exceeds HQD-only and DQE-only by 2.78 and 1.25 percentage points in Macro-F1, respectively. The class-wise maxima remain distributed across several methods, indicating that LateAvg provides the strongest aggregate balance rather than the best result for every individual class. Fusion behavior and statistical significance of the observed differences are analyzed in the following sections.

### 5.3. Complementarity and Fusion Strategy Analysis

The complementarity of HQD and DQE was evaluated by comparing the two single-branch models with representative feature-level, relation-based, gated, and decision-level integration strategies. The results are presented in Table 9.

Table 9 shows that combining HQD and DQE does not automatically improve aggregate performance. Feature concatenation and the shared-latent variants do not exceed DQE-only in mean Macro-F1, while Diff/Product improves L-F1 at the expense of weaker aggregate and High-class performance. GMU-Fusion provides the strongest feature-level fusion reference but remains slightly below LateAvg in the three aggregate metrics.

LateAvg achieves the highest mean Accuracy, Macro-F1, Weighted-F1, and M-F1 among the evaluated single-branch and fusion strategies in terms of average fold performance. These results indicate that, under the current limited-data setting, independently optimizing the two evidence branches and combining their outputs through decision-level posterior averaging provides an effective and stable integration strategy. The improvement over GMU-Fusion is relatively small, and the paired fold-wise statistical analysis is further presented in Section 5.4. Sensitivity to different fusion weights is reported in Supplementary Table S5.

### 5.4. MANIQA Adaptation and Statistical Analysis

Late-stage MANIQA fine-tuning increases Macro-F1 from 0.8385 ± 0.0516 for DQE-only to 0.8447 ± 0.0580. LateAvg achieves 0.8510 ± 0.0612 while retaining the frozen deep representation, indicating that explicit task-oriented quality evidence can provide comparable aggregate performance to partial backbone adaptation under the current limited-data setting.

Paired two-tailed *t*-tests and Wilcoxon signed-rank tests were conducted using the five fold-level Macro-F1 scores. Table 10 reports the paired differences and *p*-values, while Supplementary Table S6 provides the corresponding fold-wise results. Given the limited number of folds, these statistical analyses are interpreted as exploratory evidence rather than definitive significance tests. The Wilcoxon results should be interpreted cautiously because the small number of paired observations limits statistical resolution.

LateAvg shows a positive mean Macro-F1 difference against every listed comparator. The paired *t*-tests reach the nominal 0.05 level only for NIQE-score and TOPIQ-score, whereas Wilcoxon signed-rank tests remain non-significant for all comparisons. Therefore, the results indicate higher average performance for LateAvg, but do not establish statistically significant superiority over stronger conventional, single-branch, fusion, or fine-tuning alternatives.

Because the analysis contains only five paired fold-level observations, the statistical power is limited. LateAvg achieves higher fold-level Macro-F1 than DQE-only in four of five folds and than GMU-Fusion in three of five folds, suggesting a consistent but modest empirical advantage under the current fish-group five-fold protocol. However, the fold-wise ranking is not uniform, and larger-scale evaluations are required to further verify the observed performance differences.

### 5.5. Computational Cost and End-to-End Efficiency Analysis

To evaluate the deployment feasibility of the proposed framework, an end-to-end efficiency analysis was conducted using the complete inference pipeline from raw input image to final annotatability prediction. Unlike the cached-feature analysis, this evaluation includes all major computational stages involved in practical deployment, including eye-region localization, ROI preprocessing, HQD extraction, MANIQA-based DQE extraction, branch prediction, and LateAvg fusion. The benchmark was performed on 200 images using the same implementation environment as the main experiments. The detailed latency breakdown of each computational stage is summarized in Table 11.

The relatively large standard deviation of ROI detection latency mainly results from variations in candidate detection numbers and occasional fallback to full-image processing when no eye-region bounding box satisfies the confidence threshold.

Beyond latency and memory consumption, the parameter scale and computational complexity of the trainable components were further analyzed. The HQD branch contains 91.0 K trainable parameters and requires approximately 91.6 K MACs per inference, while the DQE branch contains 199.3 K trainable parameters and requires approximately 200.1 K MACs. The complete LateAvg classifier, including both quality-evidence branches, contains 290.3 K trainable parameters with approximately 291.7 K MACs per sample. Since MANIQA is used as a frozen feature extractor, its parameters are kept frozen and are not updated during annotatability learning; the frozen backbone contains 135.75 M parameters and requires approximately 108.3 G MACs for feature extraction from a 224 × 224 ROI.

The complete LateAvg pipeline requires approximately 373.93 ms per image under the evaluated deployment setting. Among all components, ROI detection introduces the largest computational overhead, followed by deep quality embedding extraction using MANIQA. In contrast, branch classification and decision-level posterior averaging introduce only limited additional computation, with the final fusion operation requiring less than 1 ms per image. These results indicate that the proposed decision-level integration strategy remains lightweight, while the overall runtime is mainly determined by image localization and quality-evidence extraction.

The GPU memory requirement was additionally measured during inference. Using CUDA execution on 200 benchmark images, the peak GPU memory consumption was 682.35 MB. This moderate memory footprint indicates that the proposed framework can be deployed on commonly available GPU-equipped systems for fish iris dataset construction and pre-annotation quality control.

Although the current implementation focuses on image-based pre-annotation screening rather than real-time acquisition systems, the end-to-end analysis demonstrates that LateAvg provides a practical computational cost for automated pre-annotation assessment. The results also identify the main optimization opportunities for future deployment, particularly accelerating ROI localization and deep quality feature extraction.

### 5.6. Qualitative, Complementarity, and Deployment Analysis

To characterize the behavior of LateAvg beyond aggregate performance metrics, representative prediction cases, dataset-level branch complementarity, and the row-normalized confusion matrix were examined. These analyses evaluate whether the HQD and DQE branches provide non-redundant annotatability evidence, how decision-level posterior averaging handles branch disagreements, and where the remaining classification ambiguity is concentrated.

Figure 4 illustrates the complementary roles of HQD and DQE. In Figure 4a,b, DQE-only corrects errors made by HQD-only, whereas Figure 4c,d show the converse pattern. LateAvg retains the correct prediction in these examples, indicating that the two branches provide non-redundant evidence. The failures in Figure 4e,f occur between adjacent annotatability levels and illustrate the ambiguity of samples near the class boundaries.

Figure 5 quantifies branch complementarity across all held-out samples aggregated from the five folds. As shown in Figure 5a, HQD-only and DQE-only are uniquely correct on 112 and 132 samples, respectively, confirming that neither branch fully subsumes the other; branch disagreement is most pronounced for Medium samples. The two branches disagree on 257 samples (17.1%), of which LateAvg correctly classifies 151 (58.8%), as shown in Figure 5b. These results indicate that equal-weight decision-level posterior averaging captures a substantial, but incomplete, portion of the complementary evidence, suggesting that future work may investigate adaptive fusion strategies such as confidence-aware weighting or dynamic branch selection.

Figure 6 shows that LateAvg achieves a pooled overall accuracy of 85.5% across all 1500 held-out test samples (corresponding to the per-fold mean of 0.8574 reported in Table 8), with the strongest recognition observed for High samples and the weakest for Medium samples. Most errors occur between Medium and Low, including 105 Medium-to-Low and 72 Low-to-Medium predictions. All 218 errors are confined to adjacent annotatability levels, with no direct confusion between High and Low. The remaining ambiguity is therefore concentrated primarily at the Medium–Low boundary.

Taken together, these results support LateAvg as a screening component for fish iris dataset construction. After image acquisition and eye-region selection, High samples can proceed to manual annotation, Medium samples can be retained for further review, and Low samples can be excluded or reacquired. The subsequent quality-filtering analysis further confirms that this screening strategy improves the reliability of the retained annotation pool while maintaining practical sample coverage. Therefore, LateAvg serves as an acquisition-to-annotation triage module that prioritizes annotation effort rather than replacing manual annotation.

### 5.7. Quality Filtering Validation for Annotation Resource Allocation

To evaluate the practical value of annotatability assessment for pre-annotation quality control, a quality-filtering experiment was conducted using the predictions of the LateAvg model. The objective was to examine whether model-assisted sample prioritization could improve the reliability of the retained annotation pool and enhance annotation resource utilization while maintaining reasonable sample coverage.

Three annotation strategies were compared: (1) All images, where all 1500 samples were retained without filtering; (2) Remove Low, where samples predicted as Low annotatability were excluded before annotation; and (3) High only, where only samples predicted as High annotatability were retained. The latter was included as a high-confidence reference to characterize the upper bound of reliability improvement rather than as a practical deployment strategy. The retained sample ratio was evaluated using coverage, while annotation reliability was measured by the mean Annotation Reliability Score (ARS) and mean geometric consistency (IoU).

The results are summarized in Table 12. Without filtering, the complete dataset achieved an average ARS of 0.5927 and an average IoU of 0.5977. After removing samples predicted as Low annotatability, 965 images were retained, corresponding to a coverage of 64.33%. Meanwhile, the mean ARS increased to 0.7457 and the mean IoU increased to 0.7479. Compared with the unfiltered setting, this strategy improved ARS by 25.8% and IoU by 25.1%, while preserving more than half of the original samples. In contrast, the High-only strategy further increased ARS to 0.8338 and IoU to 0.8358, but reduced the retained coverage to 34.87%.

Figure 7 illustrates the trade-off between retained coverage and annotation reliability. The results show that more aggressive filtering increases the reliability of the retained annotation pool at the cost of reduced sample coverage, revealing the trade-off between annotation reliability and available annotation resources. Removing only Low-annotatability samples provides a more balanced operating point, improving the reliability of retained samples without excessively reducing the available annotation pool.

Statistical analysis using the Mann–Whitney U test further confirmed the reliability improvement. Compared with the unfiltered dataset, the Remove Low strategy achieved significant improvements in both ARS $\left(p<0.001\right)$ and IoU $\left(p<0.001\right)$. These results indicate that annotatability assessment can provide an effective front-end screening mechanism for biological image annotation workflows. Rather than replacing expert annotation, the proposed framework assists annotation resource allocation by prioritizing samples with stronger boundary-traceability evidence and reducing unnecessary manual effort on unreliable samples.

## 6. Discussion

### 6.1. Key Findings and Practical Implication

The results confirm that fish iris annotatability cannot be reliably represented by general perceptual quality scores. Task-oriented representation-based methods exceed the strongest scalar IQA baseline by approximately 0.24 in mean Macro-F1, reflecting a fundamental mismatch between global quality assessment and local boundary traceability. This difference should not be interpreted simply as an architectural advantage. Rather, it indicates that effective quality control depends on whether the evidence representation is aligned with the target annotation decision. For fine-grained structural tasks, image usability should therefore be judged according to the visibility and traceability of the target structure rather than overall visual clarity.

The experiments also show that HQD and DQE provide complementary but heterogeneous annotatability evidence. Their disagreement patterns demonstrate that neither representation fully subsumes the other, while the fusion comparisons indicate that more complex feature coupling does not necessarily yield better aggregate performance. In this limited-data setting, independently learning the two evidence sources and combining their outputs through decision-level posterior averaging provides a stable integration strategy. The value of LateAvg therefore lies in preserving branch-specific decision information and exploiting complementarity at the decision level, rather than in increasing architectural complexity.

The three-level formulation further provides a practical basis for acquisition-to-annotation triage. High samples can proceed to routine annotation, Medium samples can be retained for further review, and Low samples can be excluded or reacquired. Beyond predictive performance, the quality-filtering analysis demonstrates that removing predicted Low-annotatability samples increases the geometric annotation reliability of the retained image pool, confirming the practical value of annotatability assessment for pre-annotation quality control. Such screening is particularly valuable for biological eye-image analysis, where reliable iris or ocular structure annotation supports downstream tasks including individual identification, behavioral analysis, and ecological monitoring. LateAvg should therefore be regarded as a front-end screening component that supports annotation resource allocation rather than as a replacement for expert delineation.

### 6.2. Limitations

Several limitations of this work should be acknowledged.

Because the current task-specific evaluation dataset includes only two freshwater fish species with relatively similar iris morphologies and was collected using a single imaging system, its external validity remains limited. Generalization to larger fish species, marine organisms, substantially different ocular structures, other imaging devices, and diverse underwater environments has not yet been established. Furthermore, public datasets specifically designed for fish iris annotatability assessment are currently unavailable. Existing fish-eye and underwater vision datasets mainly focus on recognition, detection, or segmentation tasks and generally lack the fine-grained boundary-traceability labels required for pre-annotation quality control. Therefore, the present results should be interpreted as proof-of-concept evidence for boundary-traceability-based annotatability assessment rather than as evidence of a universally applicable fish iris screening model. Future validation on larger-scale cross-species and cross-platform benchmarks, with standardized annotatability definitions and multi-annotator reliability assessment, will be necessary to further establish the generalizability of the framework.

By design, the current framework operates on individual static images and does not exploit temporal redundancy in continuous video. Adjacent frames may provide complementary boundary evidence when transient motion blur, reflection, or occlusion makes a single image difficult to assess. Moreover, although the current end-to-end analysis provides a complete inference cost estimate, further optimization of ROI localization and deep quality feature extraction remains necessary for real-time deployment.

With respect to representation coverage, the handcrafted descriptors mainly characterize intensity, illumination, sharpness, edge response, noise, and texture. They do not explicitly model all underwater-specific degradation processes, such as light scattering, chromatic distortion, and suspended-particle interference. In addition, only frozen MANIQA embeddings were evaluated as the deep quality representation, and systematic comparisons with other pretrained image-quality backbones remain necessary.

Although fish-group five-fold cross-validation reduces identity-level leakage and evaluates generalization to unseen fish individuals, the resulting five-fold-level observations provide limited statistical power for distinguishing closely performing methods. The paired tests reported in Section 5.4 should therefore be interpreted as exploratory rather than as definitive evidence of superiority.

Although the quality-filtering experiment demonstrates improved geometric annotation reliability, the direct reduction of manual annotation time and cost was not quantitatively measured in this study. Future work will evaluate annotation workload reduction through controlled human annotation experiments.

Label reproducibility constitutes a further limitation. The operational H/M/L grades were assigned by a single domain-experienced annotator rather than through multi-assessor consensus, while the three additional annotators contributed geometric iris annotations for ARS-based reliability modeling but did not independently assign H/M/L labels. Consequently, the reported performance reflects consistency with one expert’s grading standard, and transferability to criteria established by other annotators or annotation teams remains uncertain. Independent multi-assessor grading on a representative subset, together with inter-rater agreement analysis such as Cohen’s or Fleiss’ kappa, is needed to establish label reproducibility and distinguish method effects from rater effects.

### 6.3. Future Research Directions

Future work should extend evaluation through larger-scale cross-species and cross-platform benchmarks for fish iris annotatability assessment, followed by validation on broader biological eye-image scenarios including additional fish species, other animal ocular structures, and diverse acquisition environments. Since the proposed framework focuses on boundary traceability rather than species-specific biological characteristics, the underlying annotatability assessment principle may be applicable to other fine-grained biological image annotation tasks where structural delineation reliability is critical. Temporal information from video sequences may provide additional evidence for borderline samples, and confidence-aware fusion weighting or dynamic branch selection may improve the handling of branch disagreements. Future work should further validate the framework in larger-scale annotation workflows by investigating its effects on annotation workload, inter-annotator consistency, reacquisition decisions, and downstream segmentation or annotation efficiency.

## 7. Conclusions

This study recasts fish iris image quality control as a task-conditioned annotatability assessment problem, where the key criterion is not overall visual quality but the traceability of relevant iris boundaries by human annotators. By integrating explicit boundary-related quality evidence with learned deep quality representations, the proposed framework provides a practical approach for pre-annotation quality control. Under a fish-group five-fold protocol on 1500 self-collected images, the framework achieves 0.8574 accuracy and 0.8510 Macro-F1. The results demonstrate that integrating heterogeneous quality evidence through a lightweight decision-level posterior averaging strategy provides an effective assessment approach under the current limited-data setting, although statistical superiority over stronger baselines is not established.

Beyond predictive performance, the quality-filtering analysis indicates that annotatability assessment can improve the reliability of retained annotation samples while supporting more efficient allocation of manual annotation resources. Future work will investigate broader applicability across biological eye-image tasks, multi-assessor label reproducibility, and large-scale annotation workflows to further establish the generalizability of the boundary-traceability-based assessment paradigm.

## Figures and Tables

**Figure 1:**
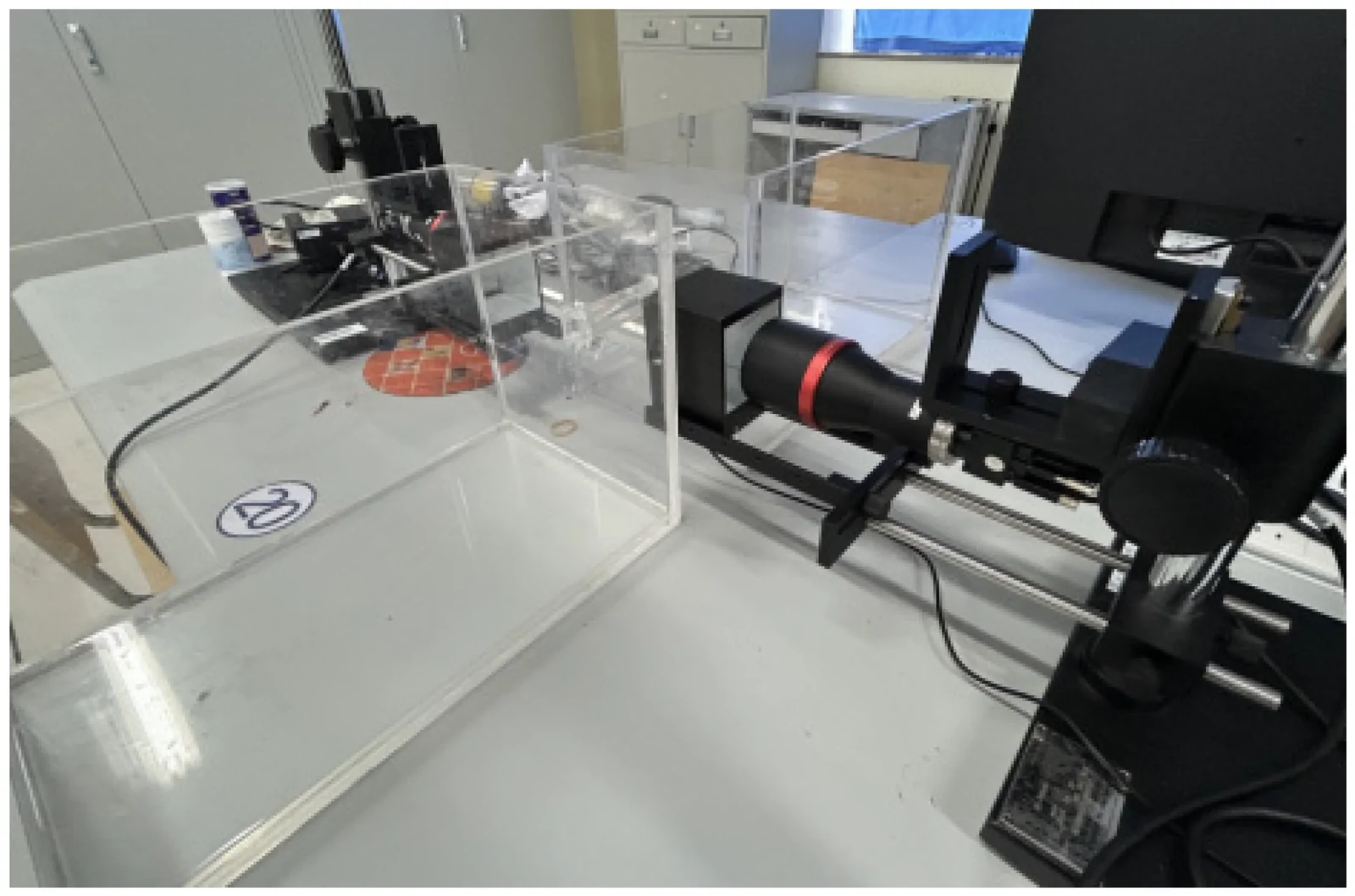
Underwater non-contact fish iris image acquisition system used for dataset construction.

**Figure 2:**
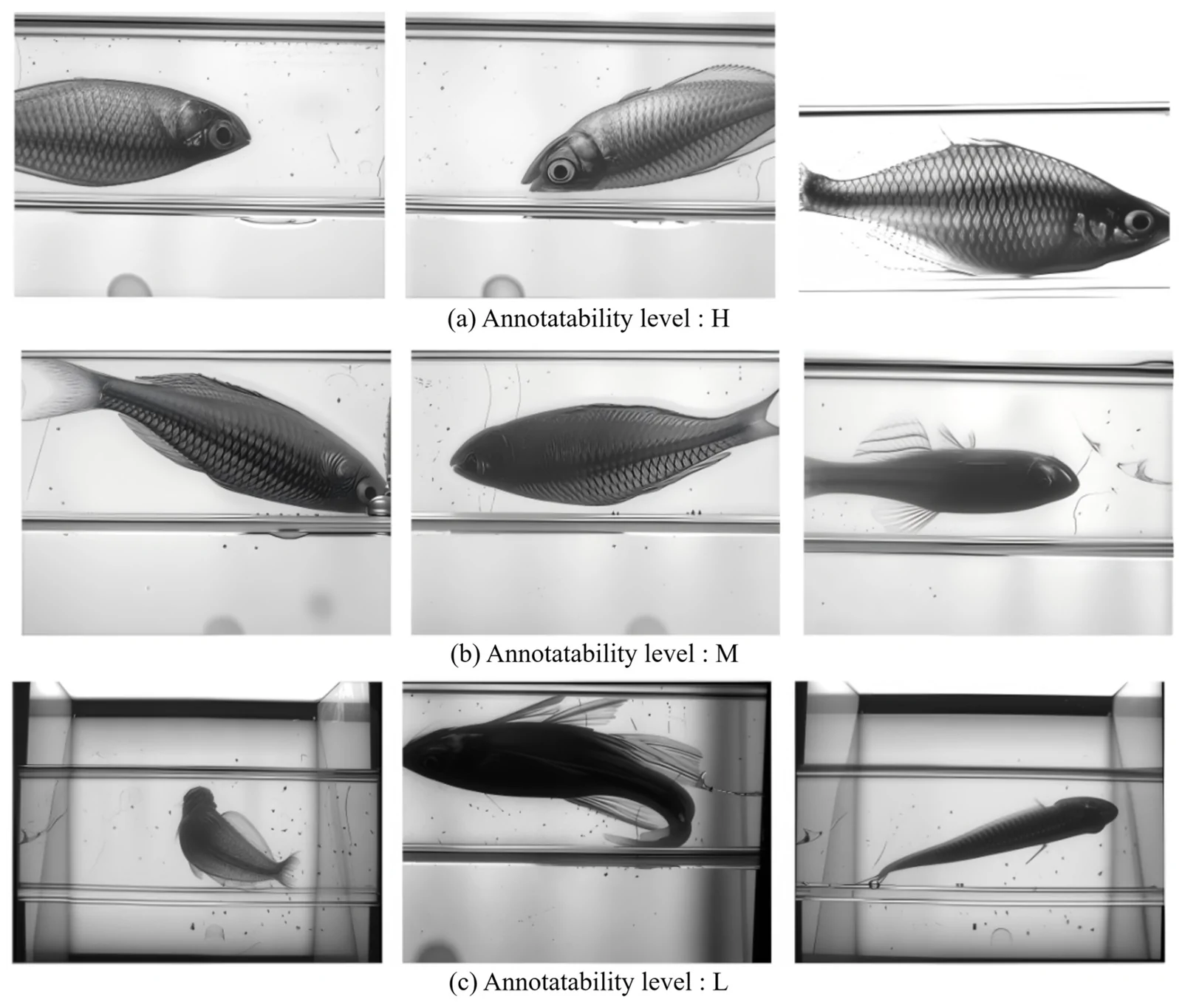
Representative full-body fish images with different fish iris annotatability levels. The annotatability level is determined by whether the eye/iris region provides sufficient visual evidence for reliable inner- and outer-boundary delineation.

**Figure 3:**
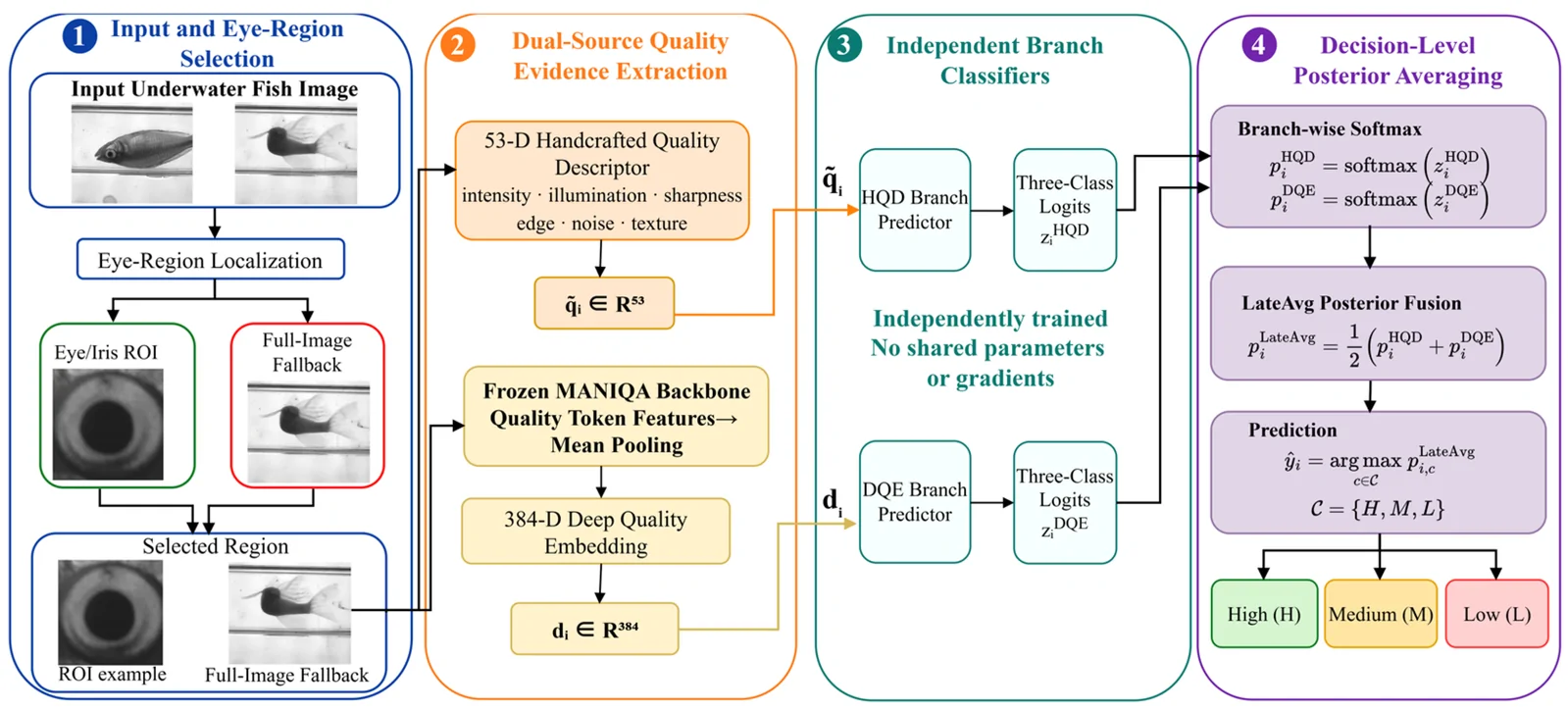
Overview of the proposed LateAvg framework for fish iris image annotatability assessment. Two heterogeneous quality representations, including handcrafted quality descriptors and frozen MANIQA-based deep quality embeddings, are independently classified and integrated through equal-weight decision-level posterior averaging.

**Figure 4:**
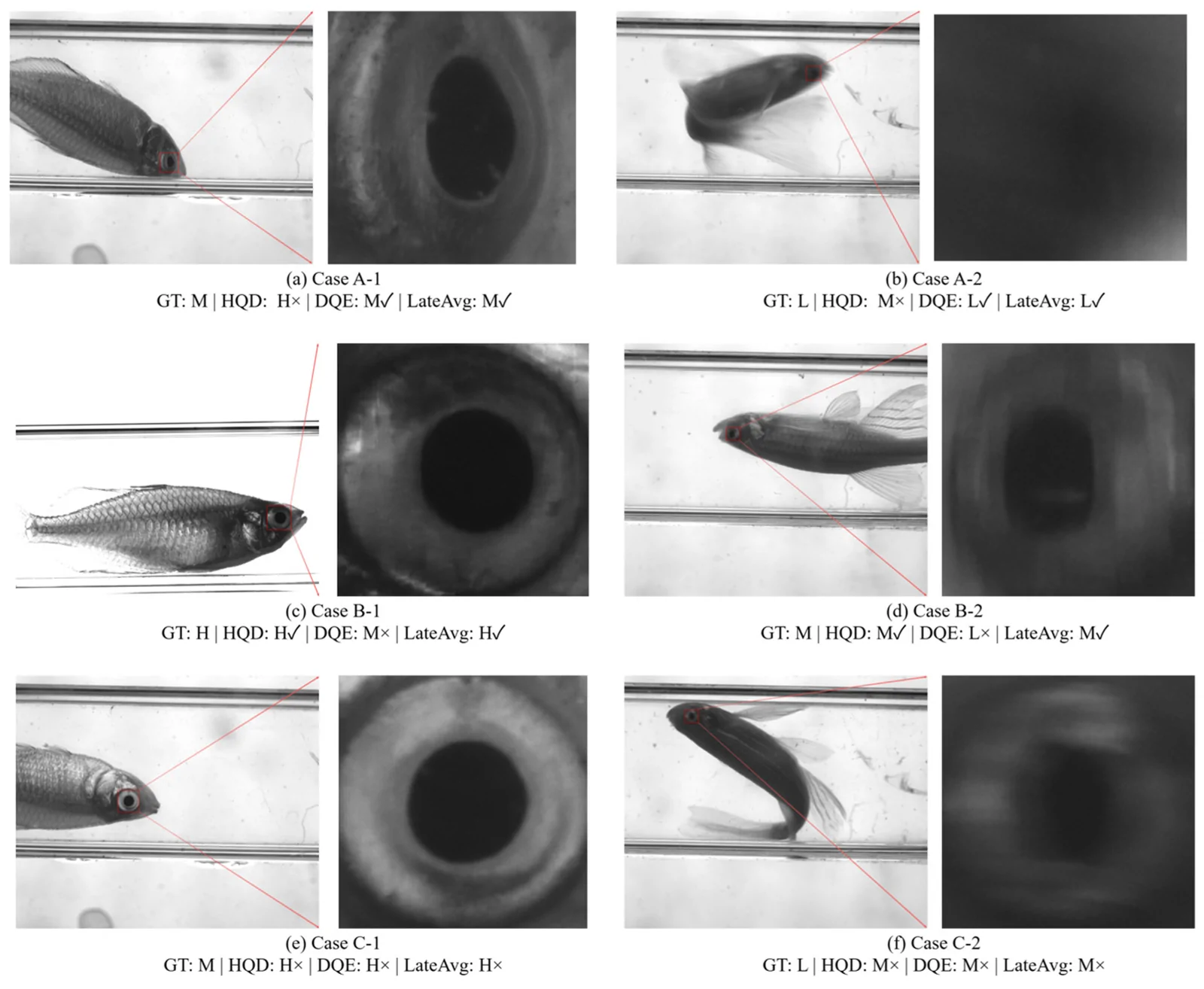
Representative complementary and failure cases of LateAvg. Each panel presents the original image and the corresponding eye-region crop. Panels (**a**,**b**) show cases in which DQE-only is correct while HQD-only fails, whereas panels (**c**,**d**) show the converse cases. Panels (**e**,**f**) present representative LateAvg errors. H, M, and L denote High, Medium, and Low annotatability, respectively; ✓ and × indicate correct and incorrect predictions.

**Figure 5:**
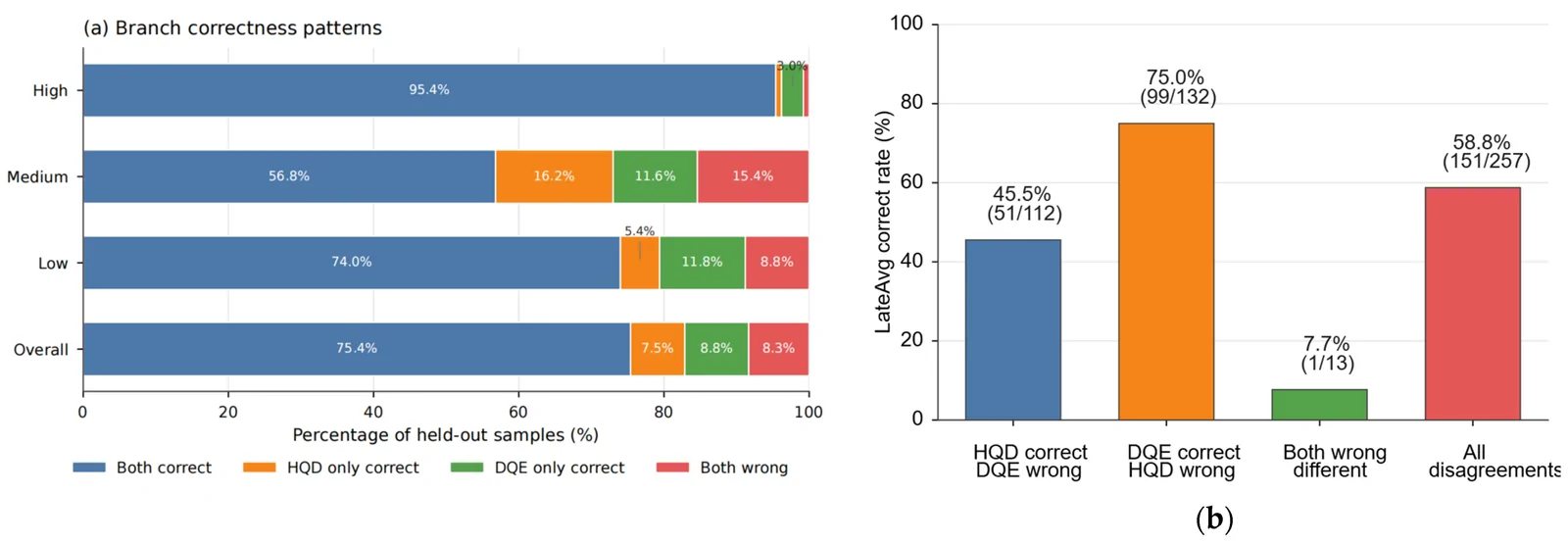
Branch complementarity and LateAvg outcomes on the aggregated held-out samples. (**a**) Branch correctness patterns of HQD-only and DQE-only across all held-out samples, summarized by true annotatability class and overall. (**b**) LateAvg correct rates on samples for which HQD-only and DQE-only produced different predictions, grouped by branch-correctness pattern and reported for the complete disagreement subset.

**Figure 6:**
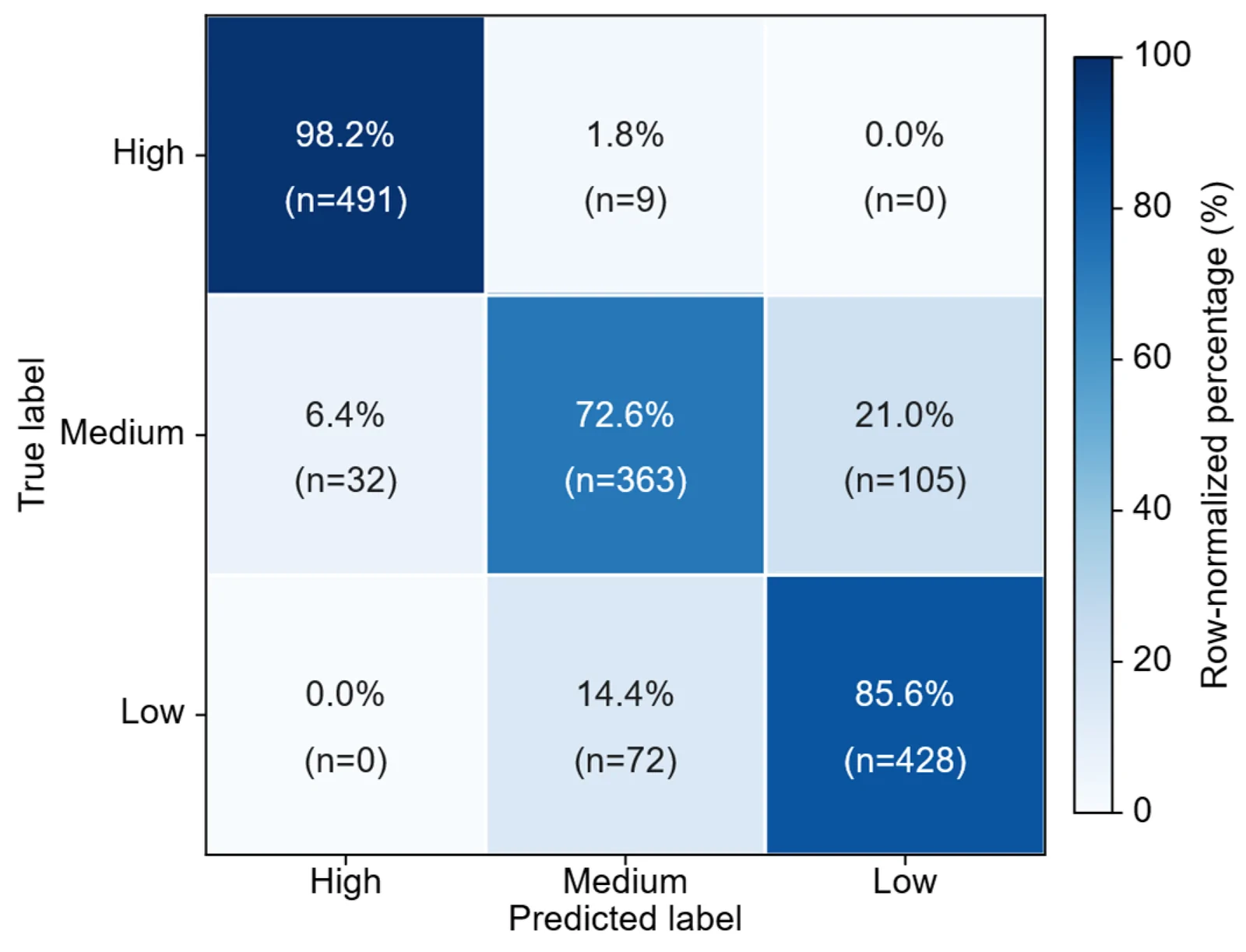
Row-normalized confusion matrix of LateAvg on the aggregated held-out samples. Each cell reports the row-normalized percentage and sample count for the corresponding true-and-predicted label pair.

**Figure 7:**
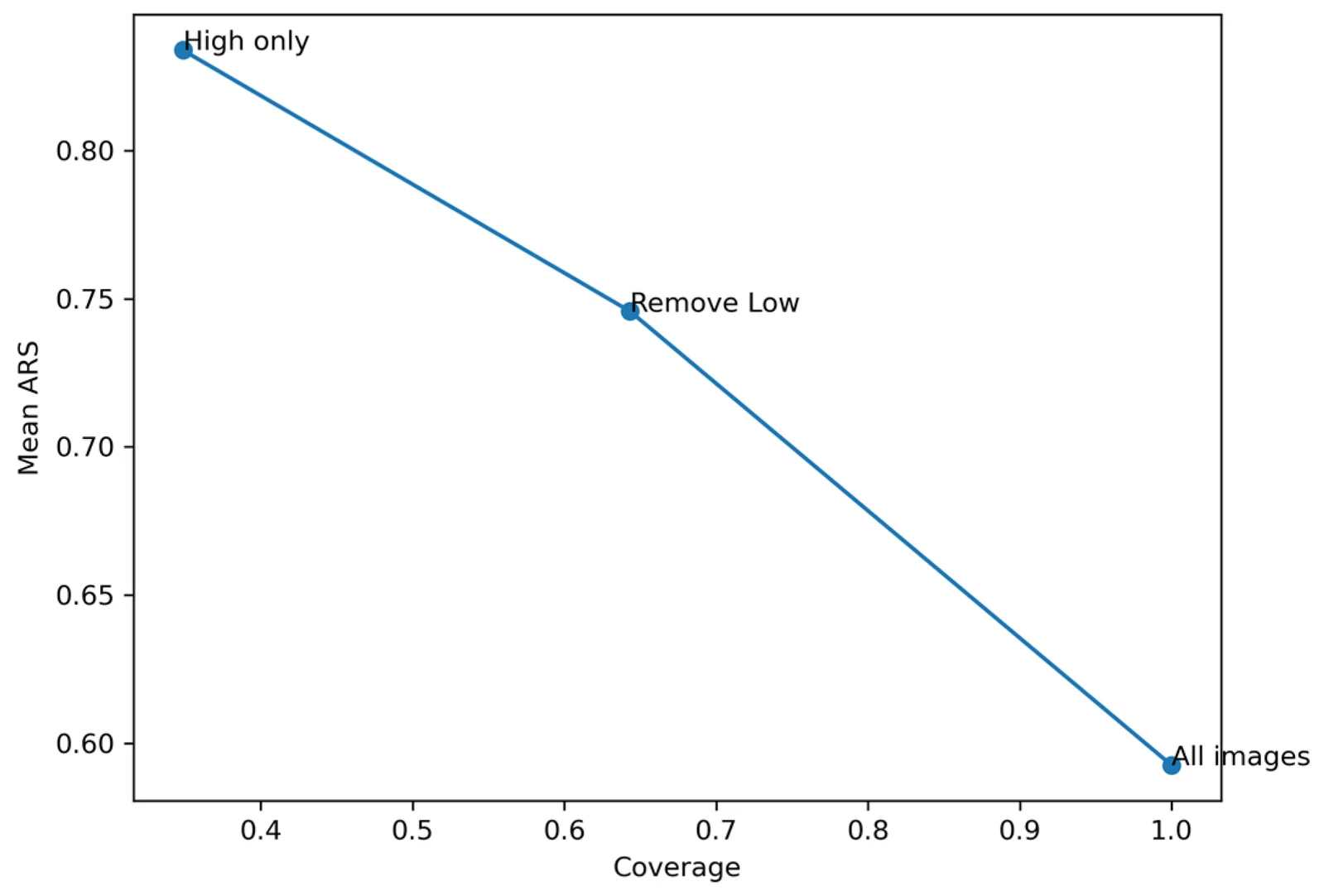
Coverage–quality trade-off under different quality-filtering strategies. The x-axis represents the retained sample coverage, and the y-axis represents the mean Annotation Reliability Score (ARS). The results demonstrate the trade-off between retaining more samples and improving annotation reliability.

**Table 1:** Definition of fish iris image annotatability levels.

Level	Definition	Visual Criteria	Annotation Decision
High (H)	The iris region provides sufficient visual evidence for reliable annotation.	The fish eye region is clearly visible; the iris structure is distinguishable; the inner and outer iris boundaries are relatively clear; and blur, reflection, or occlusion is minimal.	Suitable for direct manual annotation.
Medium (M)	The iris region remains partially annotatable but contains uncertainty.	The fish eye/iris region is visible, but partial blur, reflection, low contrast, occlusion, or boundary ambiguity may affect the stability of inner- and outer-boundary delineation.	Requires further human review or annotation assistance before formal annotation.
Low (L)	The iris region lacks sufficient visual evidence for reliable annotation.	The fish eye region is missing, severely blurred, occluded, underexposed, strongly reflected, or indistinguishable; the inner and outer iris boundaries cannot be reliably identified.	Exclusion or reacquisition.

**Table 2:** Statistics of geometric annotation reliability.

Item	Value
Images with 3 valid annotations	1287
Images with 2 valid annotations	68
Images with 1 valid annotation	38
Images with 0 valid annotations	107
$\mathrm{Mean}\;(\bar{IoU})$	0.5977
$\mathrm{Min}/\mathrm{Max}\;(\bar{IoU})$	0.0000/0.9392
$\mathrm{Mean}\;({ARS}^{norm}$)	0.4643
$\mathrm{Min}/\mathrm{Max}\;({ARS}^{norm}$)	0.0000/1.0000

**Table 3:** Fish-group five-fold data split statistics.

Fold	Train Samples	Validation Samples	Test Samples	H/M/L in Test Set
Fold 0	981	192	327	99/137/91
Fold 1	997	221	282	120/100/62
Fold 2	868	289	343	131/100/112
Fold 3	1016	222	262	87/75/100
Fold 4	962	252	286	63/88/135

**Table 4:** Composition of the 53-dimensional Handcrafted Quality Descriptor.

Feature Group	Dim.	Representative Descriptors	Annotatability Relevance
Gray-level intensity statistics	10	Mean, standard deviation, percentiles, dynamic range, entropy, skewness, and kurtosis	Intensity distribution and exposure stability
HSV color and illumination statistics	12	Hue, saturation, and value statistics; bright, dark, and low-saturation ratios	Illumination quality and local visibility
Sharpness, edge, and gradient statistics	12	Laplacian, gradient magnitude, edge density, Tenengrad, and high-frequency energy	Boundary clarity and contour reliability
Noise and local variation	6	Residual statistics, local variance, and signal-to-residual ratio	Noise level and local instability
GLCM texture and structure	8	Contrast, dissimilarity, homogeneity, energy, correlation, and entropy	Texture regularity and structural distinguishability
Gabor directional texture	5	Directional Gabor responses and response variation	Directional structure and local pattern visibility

**Table 5:** Main experimental settings.

Category	Setting	Value
Dataset	Images and classes	1500 images; High, Medium, and Low
Evaluation protocol	Data partitioning	Fish-group five-fold cross-validation
Preprocessing	Fold-dependent statistics	Estimated from the training split only
Input representation	Explicit quality evidence	53-dimensional HQD
Input representation	Implicit quality evidence	384-dimensional DQE from frozen MANIQA
Model architecture	MLP classifier	Linear-LayerNorm-GELU-Dropout MLP classifier (dropout = 0.2); 128-dimensional hidden representation
Fusion	LateAvg	Equal-weight posterior averaging
Supervision	Neural models	Reliability-aware soft targets and sample weights
Supervision	Scalar-IQA and conventional-ML baselines	Manual hard labels without sample weighting
Optimization	Optimizer	AdamW
Optimization	Learning rate/weight decay	$1\times 10^{-4}/1\times 10^{-4}$
Hardware	GPU	NVIDIA GeForce RTX 4050 Laptop GPU
Software	Framework	PyTorch 2.8.0, CUDA 12.8
Training	Batch size/maximum epochs	64/50
Training	Early stopping/gradient clipping	10 epochs/1.0

**Table 6:** Performance of IQA final-score baselines under five-fold cross-validation.

Category	Method	Accuracy	Macro-F1	Weighted-F1	H-F1	M-F1	L-F1
Traditional NR-IQA	BRISQUE-score	0.4031 ± 0.0595	0.3533 ± 0.0745	0.3543 ± 0.0812	0.5294 ± 0.0897	0.1892 ± 0.2595	0.3414 ± 0.2117
Traditional NR-IQA	NIQE-score	**0.6303 ± 0.0890**	**0.6139 ± 0.1003**	**0.6146 ± 0.1195**	**0.7253 ± 0.0658**	0.4740 ± 0.1936	**0.6423 ± 0.1556**
Traditional NR-IQA	PIQE-score	0.4361 ± 0.0530	0.3700 ± 0.0626	0.3632 ± 0.0598	0.0963 ± 0.2153	0.5607 ± 0.0913	0.4530 ± 0.1190
Underwater IQA	UIQM-score	0.4500 ± 0.1412	0.4176 ± 0.0933	0.4171 ± 0.1102	0.5201 ± 0.1856	0.6048 ± 0.1498	0.1280 ± 0.0694
Underwater IQA	UCIQE-score	0.4377 ± 0.1651	0.3978 ± 0.1141	0.3899 ± 0.1348	0.5443 ± 0.1903	0.5592 ± 0.2281	0.0898 ± 0.0580
Deep IQA	MANIQA-score	0.4876 ± 0.0773	0.4369 ± 0.0795	0.4318 ± 0.1042	0.3210 ± 0.2243	0.3584 ± 0.2323	0.6312 ± 0.1271
Deep IQA	MUSIQ-score	0.4980 ± 0.0555	0.4836 ± 0.0554	0.4793 ± 0.0766	0.5290 ± 0.1687	0.6602 ± 0.0317	0.2616 ± 0.0681
Deep IQA	DBCNN-score	0.5131 ± 0.0799	0.5069 ± 0.0769	0.5061 ± 0.0908	0.6561 ± 0.0790	0.5109 ± 0.1354	0.3537 ± 0.0769
Deep IQA	TOPIQ-score	0.6048 ± 0.0556	0.5877 ± 0.0508	0.6020 ± 0.0574	0.6508 ± 0.0941	**0.7120 ± 0.0627**	0.4002 ± 0.1503
Aesthetic IQA	NIMA-score	0.4439 ± 0.0847	0.4346 ± 0.0743	0.4372 ± 0.0883	0.5062 ± 0.1487	0.6102 ± 0.0694	0.1874 ± 0.0426

Note: Bold values indicate the best performance among the compared methods.

**Table 7:** Performance of conventional 53D HQD baselines under five-fold cross-validation.

Method	Input	Accuracy	Macro-F1	Weighted-F1	H-F1	M-F1	L-F1
RF-53D	53D HQD	0.7931 ± 0.1179	0.7842 ± 0.1159	0.7914 ± 0.1166	0.9036 ± 0.0689	0.7127 ± 0.1277	0.7362 ± 0.2094
XGBoost-53D	53D HQD	0.8070 ± 0.1139	0.7975 ± 0.1133	0.8049 ± 0.1114	0.9086 ± 0.0675	0.7216 ± 0.1345	0.7622 ± 0.1962
LR-53D	53D HQD	0.8322 ± 0.0747	**0.8234 ± 0.0725**	**0.8306 ± 0.0735**	**0.9168 ± 0.0893**	**0.7720 ± 0.0884**	0.7813 ± 0.1068
SVM-53D	53D HQD	**0.8323 ± 0.0897**	0.8188 ± 0.0982	0.8296 ± 0.0916	0.9099 ± 0.1312	0.7498 ± 0.1410	**0.7966 ± 0.1245**

Note: Bold values indicate the best performance among the compared methods.

**Table 8:** Main comparison under fish-group five-fold cross-validation.

Comparison Method Category	Method	Evidence Source	Accuracy	Macro-F1	Weighted-F1	H-F1	M-F1	L-F1
Scalar IQA	NIQE-score	1D traditional IQA score	0.6303 ± 0.0890	0.6139 ± 0.1003	0.6146 ± 0.1195	0.7253 ± 0.0658	0.4740 ± 0.1936	0.6423 ± 0.1556
Scalar IQA	TOPIQ-score	1D deep IQA score	0.6048 ± 0.0556	0.5877 ± 0.0508	0.6020 ± 0.0574	0.6508 ± 0.0941	0.7120 ± 0.0627	0.4002 ± 0.1503
Conventional ML	LR-53D	53D handcrafted descriptors	0.8322 ± 0.0747	0.8234 ± 0.0725	0.8306 ± 0.0735	0.9168 ± 0.0893	**0.7720 ± 0.0884**	0.7813 ± 0.1068
Single branch	HQD-only	53D HQD	0.8327 ± 0.0786	0.8232 ± 0.0800	0.8293 ± 0.0802	0.9208 ± 0.0653	0.7465 ± 0.1269	0.8022 ± 0.1174
Single branch	DQE-only	Frozen MANIQA384 embedding	0.8446 ± 0.0560	0.8385 ± 0.0516	0.8408 ± 0.0601	**0.9595 ± 0.0206**	0.7403 ± 0.1105	0.8156 ± 0.0641
Fusion reference	GMU-Fusion	53D + MANIQA384 features	0.8536 ± 0.0516	0.8462 ± 0.0512	0.8489 ± 0.0565	0.9443 ± 0.0351	0.7579 ± 0.1071	**0.8366 ± 0.0591**
Deep reference	MANIQA- LateStage-FT	ROI/fallback image	0.8526 ± 0.0560	0.8447 ± 0.0580	0.8495 ± 0.0607	0.9459 ± 0.0257	0.7526 ± 0.1270	0.8356 ± 0.0811
Proposed	LateAvg (Ours)	HQD + DQE posteriors	**0.8574 ± 0.0638**	**0.8510 ± 0.0612**	**0.8541 ± 0.0671**	0.9549 ± 0.0357	0.7685 ± 0.1181	0.8296 ± 0.0757

Note: Bold values indicate the best performance among the compared methods.

**Table 9:** Comparison of single-branch and fusion strategies.

Method	Fusion Strategy	Accuracy	Macro-F1	Weighted-F1	H-F1	M-F1	L-F1
HQD-only	Explicit single branch	0.8327 ± 0.0786	0.8232 ± 0.0800	0.8293 ± 0.0802	0.9208 ± 0.0653	0.7465 ± 0.1269	0.8022 ± 0.1174
DQE-only	Implicit single branch	0.8446 ± 0.0560	0.8385 ± 0.0516	0.8408 ± 0.0601	**0.9595 ± 0.0206**	0.7403 ± 0.1105	0.8156 ± 0.0641
Concat	Feature concatenation	0.8433 ± 0.0822	0.8337 ± 0.0844	0.8388 ± 0.0876	0.9315 ± 0.0734	0.7450 ± 0.1486	0.8246 ± 0.0709
Diff/Product	Relation feature fusion	0.8475 ± 0.0671	0.8398 ± 0.0710	0.8438 ± 0.0720	0.9219 ± 0.0553	0.7557 ± 0.1383	**0.8419 ± 0.0623**
Full w/o Gate	Shared-latent fusion	0.8340 ± 0.0609	0.8269 ± 0.0606	0.8299 ± 0.0645	0.9423 ± 0.0410	0.7376 ± 0.1009	0.8008 ± 0.0868
GMU-Fusion	Feature-wise gated fusion	0.8536 ± 0.0516	0.8462 ± 0.0512	0.8489 ± 0.0565	0.9443 ± 0.0351	0.7579 ± 0.1071	0.8366 ± 0.0591
Shared-latent gated fusion	Scalar-gated latent fusion	0.8392 ± 0.0653	0.8318 ± 0.0654	0.8338 ± 0.0708	0.9368 ± 0.0335	0.7390 ± 0.1276	0.8194 ± 0.0839
LateAvg (Ours)	Decision-level posterior averaging	**0.8574 ± 0.0638**	**0.8510 ± 0.0612**	**0.8541 ± 0.0671**	0.9549 ± 0.0357	**0.7685 ± 0.1181**	0.8296 ± 0.0757

Note: Bold values indicate the best performance among the compared methods.

**Table 10:** Paired comparison of five-fold Macro-F1 between LateAvg and representative comparators.

Comparator	Mean Difference (LateAvg − Comparator)	Wins/Ties/Losses	Paired (*t*)-Test (*p*)	Wilcoxon (*p*)
NIQE-score	+0.2371	5/0/0	0.0074	0.0625
TOPIQ-score	+0.2633	5/0/0	0.0004	0.0625
LR-53D	+0.0276	4/0/1	0.0957	0.1250
HQD-only	+0.0278	3/0/2	0.1905	0.3125
DQE-only	+0.0125	4/0/1	0.0647	0.1250
GMU-Fusion	+0.0048	3/0/2	0.7623	0.8125
MANIQA-LateStage-FT	+0.0063	3/0/2	0.8086	0.8125

**Table 11:** End-to-end inference latency breakdown of the LateAvg framework.

Stage	Latency (ms/Image)
ROI detection	174.69 ± 296.32
ROI cropping and preprocessing	27.33 ± 9.50
HQD extraction	69.96 ± 31.61
MANIQA-based DQE extraction	93.09 ± 36.20
HQD branch classification	4.55 ± 7.84
DQE branch classification	3.48 ± 3.02
LateAvg fusion	0.21 ± 0.07
Total inference time	373.93 ± 351.52

**Table 12:** Quality filtering performance under different annotation strategies.

Strategy	Retained Images	Coverage	ARS	IoU
All images	1500	100%	0.5927 ± 0.2800	0.5977 ± 0.2772
Remove Low	965	64.33%	0.7457 ± 0.1490	0.7479 ± 0.1477
High only	523	34.87%	0.8338 ± 0.0571	0.8358 ± 0.0509

## Data Availability

The raw underwater fish iris images generated in this study are not publicly available due to ongoing intellectual property protection and institutional data management restrictions. The data are available from the corresponding author upon reasonable request, subject to institutional approval. The aggregate results and supporting methodological information are provided in the article and its Supplementary Materials.

## Supplementary Materials

The following supporting information can be downloaded at: https://www.mdpi.com/article/10.3390/s26196053/s1, The following supporting information is available with this article: Table S1: Key specifications of the underwater non-contact fish iris image acquisition system; Table S2: Auxiliary information associated with each fish iris annotatability sample; Table S3: Configuration of the fixed eye-region localization module; Table S4: Detailed definitions of the 53-dimensional handcrafted quality descriptor; Note S1: Reliability-aware soft-target and sample-weight construction; Table S5: Sensitivity of LateAvg to fixed posterior fusion weights; Table S6: Fold-wise Macro-F1 scores of LateAvg and strong representation, fusion, and fine-tuning baselines. Table S7: Hyperparameter configurations of competing methods.

## Abbreviations

The following abbreviations are used in this manuscript:

ARRIVE	Animal Research: Reporting of In Vivo Experiments
ARS	Annotation Reliability Score
CNN	Convolutional Neural Network
DQE	Deep Quality Embedding
FT	Fine-Tuning
GLCM	Gray-Level Co-Occurrence Matrix
GMU	Gated Multimodal Unit
H, M, and L	High, Medium, and Low annotatability levels
HQD	Handcrafted Quality Descriptor
HSV	Hue, Saturation, and Value
IQA	Image Quality Assessment
IoU	Intersection over Union
LR	Logistic Regression
MACs	Multiply–Accumulate Operations
MANIQA	Multi-Dimension Attention Network for No-Reference Image Quality Assessment
ML	Machine Learning
MLP	Multilayer Perceptron
NR-IQA	No-Reference Image Quality Assessment
RF	Random Forest
ROI	Region of Interest
SVM	Support Vector Machine
XGBoost	Extreme Gradient Boosting
YOLO	You Only Look Once
