# Supplementary material for: Three-Level Fish Iris Image Annotatability Assessment for Pre-Annotation Quality Control Based on Boundary Traceability
Source: Sensors (Basel). 2026 Sep 24;26(19):6053. doi: 10.3390/s26196053 (PMC13667597; doi:10.3390/s26196053)
Supplement: Supplementary file 1 [file sensors-26-06053-s001.zip › sensors-4470182-supplementary.pdf]

Supplementary Materials

Fish Iris Image Annotatability Assessment via Independent Quality Evidence Learning and Posterior Averaging

Z.Y., Y.L., Y.W.

These supplementary materials provide additional information on the image-acquisition system, sample metadata, eye-region localization, handcrafted feature definitions, reliability-aware supervision, fusion-weight sensitivity, and fold-wise experimental results.

Supplementary Table S1. Key specifications of the underwater non-contact fish iris image acquisition system.

This table summarizes the main hardware and imaging parameters of the self-built underwater non-contact fish iris image acquisition system used for dataset construction.

Category	Component / Parameter	Specification
Aquarium structure	Imaging configuration	Dual-tank aquarium with transparent connecting channel
Channel	Material	PMMA acrylic
Channel	Size	27 mm × 27 mm × 86 mm
Lens	Model	BT-2336 telecentric lens
Lens	Magnification	0.243
Lens	Object field of view	Φ45.27 mm
Lens	Image field of view	Φ11 mm
Lens	Working distance	103.5 mm ± 3%
Lens	Aperture	8
Lens	Depth of field	6 mm
Lens	Distortion	< 0.08%
Lens	Telecentricity	< 0.04°
Camera	Model	MV-HS510GM industrial camera
Camera	Resolution and frame rate	2448 × 2048 pixels, up to 20 fps
Camera	Sensor type	CMOS
Light source	Model	MV-RC90W coaxial light source
Light source	Luminous area	90 mm
Control platform	Acquisition control	Computer-controlled image acquisition platform

Supplementary Table S2. Auxiliary information associated with each fish iris annotatability sample.

This table summarizes the auxiliary fields recorded for each sample, including image identity, fish identity, annotatability label, reliability information, ROI/fallback metadata, quality evidence representations, and split information.

Category	Information	Purpose
Image identity	Image identifier and image path	Basic sample indexing and data loading
Fish identity	Fish-level identity or acquisition group	Group-aware data splitting to reduce leakage
Annotatability label	Hard H/M/L label, stored as manual_label	Ground-truth target for evaluation
Reliability information	Annotation Reliability Score, normalized reliability score, reliability-aware soft label, and sample weight	Reliability-aware deep model training and annotation uncertainty analysis
Region metadata	Eye-region ROI information, selected region $\mathbf{R}_i$, and binary ROI/fallback indicator m_i	Consistent region-level input selection and fallback indication
Handcrafted quality evidence	53-dimensional Handcrafted Quality Descriptors (HQD)	Explicit and interpretable task-related quality representation
Deep quality evidence	384-dimensional frozen MANIQA-based Deep Quality Embedding (DQE)	High-level quality-aware representation
Split information	Training, validation, and test assignment under fish-group five-fold cross-validation	Reproducible model evaluation

Supplementary Table S3. Configuration of the Fixed Eye-Region Localization Module

The fixed detector was used only for offline ROI generation and was not jointly optimized with any annotatability assessment model.

Parameter	Value
Detector framework	Ultralytics YOLOv8, version 8.4.69
Detector model	YOLOv8n
Initialization	Pretrained yolov8n.pt
Detection target	Complete eye region
Data overlap with the main-task dataset	None at the image or fish-identity level
Input size	640 × 640
Training epochs	50
Batch size	16
Checkpoint selection criterion	Validation mAP@0.5:0.95
Raw inference confidence threshold	0.01
ROI acceptance threshold	0.25
High-confidence threshold	0.50
Multiple-detection rule	Highest-confidence eye-region box

Parameter	Value
Bounding-box enlargement	None
Fallback input	Full original image
Main-task ROI availability	1,400/1,500 (93.33%)
Main-task full-image fallback	100/1,500 (6.67%)

Supplementary Table S4. Detailed definitions of the 53-dimensional handcrafted quality descriptor.

Let I denote the normalized grayscale image region with N pixels. H, S, and V denote the hue, saturation, and value channels in the HSV color space. $P_q(.)$ denotes the q-th percentile, and ϵ is a small constant for numerical stability. Other symbols are defined in the table when first used.

No.	Feature ID	Feature group	Descriptor	Definition / formulation
1	feature_000	Gray-level intensity statistics	Gray mean	$\mu_I=\frac{1}{N}\sum_{p=1}^{N} I_p$
2	feature_001	Gray-level intensity statistics	Gray standard deviation	$\sigma_I=\sqrt{\frac{1}{N}\sum_{p=1}^{N}(I_p-\mu_I)^2}$
3	feature_002	Gray-level intensity statistics	Gray minimum	$\min(I)$
4	feature_003	Gray-level intensity statistics	Gray maximum	$\max(I)$
5	feature_004	Gray-level intensity statistics	Gray 5th percentile	$P_5(I)$
6	feature_005	Gray-level intensity statistics	Gray 95th percentile	$P_{95}(I)$
7	feature_006	Gray-level intensity statistics	Gray dynamic range	$P_{95}(I)-P_5(I)$
8	feature_007	Gray-level intensity statistics	Gray histogram entropy	$-\sum_b \mathrm{h}_b \log(\mathrm{h}_b+\epsilon)$, where h_b is the normalized grayscale histogram
9	feature_008	Gray-level intensity statistics	Gray skewness	$\frac{1}{N}\sum_{p=1}^{N}\left(\frac{I_p-\mu_I}{\sigma_I+\epsilon}\right)^3$
10	feature_009	Gray-level intensity statistics	Gray kurtosis	$\frac{1}{N}\sum_{p=1}^{N}\left(\frac{I_p-\mu_I}{\sigma_I+\epsilon}\right)^4$
11	feature_010	HSV color / illumination statistics	Hue mean	$\mu_H=\frac{1}{N}\sum_{p=1}^{N} H_p$
12	feature_011	HSV color / illumination statistics	Hue standard deviation	$\sigma_H=\sqrt{\frac{1}{N}\sum_{p=1}^{N}(H_p-\mu_H)^2}$

No.	Feature ID	Feature group	Descriptor	Definition / formulation
13	feature_012	HSV color / illumination statistics	Saturation mean	$\mu_S = \frac{1}{N}\sum_{p=1}^{N} S_p$
14	feature_013	HSV color / illumination statistics	Saturation standard deviation	$\sigma_S = \sqrt{\frac{1}{N}\sum_{p=1}^{N}(S_p - \mu_S)^2}$
15	feature_014	HSV color / illumination statistics	Value mean	$\mu_V = \frac{1}{N}\sum_{p=1}^{N} V_p$
16	feature_015	HSV color / illumination statistics	Value standard deviation	$\sigma_V = \sqrt{\frac{1}{N}\sum_{p=1}^{N}(V_p - \mu_V)^2}$
17	feature_016	HSV color / illumination statistics	Value 5th percentile	$P_5(V)$
18	feature_017	HSV color / illumination statistics	Value 95th percentile	$P_{95}(V)$
19	feature_018	HSV color / illumination statistics	Value dynamic range	$P_{95}(V) - P_5(V)$
20	feature_019	HSV color / illumination statistics	Over-bright pixel ratio	$\frac{1}{N}\sum_{p=1}^{N} \mathrm{I}\left(V_p > 0.98\right)$
21	feature_020	HSV color / illumination statistics	Under-dark pixel ratio	$\frac{1}{N}\sum_{p=1}^{N} \mathrm{I}\left(V_p < 0.02\right)$
22	feature_021	HSV color / illumination statistics	Low-saturation pixel ratio	$\frac{1}{N}\sum_{p=1}^{N} \mathrm{I}\left(S_p < 0.08\right)$
23	feature_022	Sharpness / edge / gradient	Mean absolute Laplacian	$\frac{1}{N}\sum_{p=1}^{N}$
24	feature_023	Sharpness / edge / gradient	Laplacian variance	$\mathrm{Var}(L)$
25	feature_024	Sharpness / edge / gradient	Mean squared Laplacian	$\frac{1}{N}\sum_{p=1}^{N} L_p^2$
26	feature_025	Sharpness / edge / gradient	Gradient magnitude mean	$\mu_M = \frac{1}{N}\sum_{p=1}^{N} M_p$
27	feature_026	Sharpness / edge / gradient	Gradient magnitude standard deviation	$\sigma_M = \sqrt{\frac{1}{N}\sum_{p=1}^{N}(M_p - \mu_M)^2}$

No.	Feature ID	Feature group	Descriptor	Definition / formulation
28	feature_027	Sharpness / edge / gradient	Gradient magnitude 90th percentile	$P_{90}(M)$
29	feature_028	Sharpness / edge / gradient	Mean squared gradient magnitude	$\frac{1}{N}\sum_{p=1}^{N} M_p^2$
30	feature_029	Sharpness / edge / gradient	Canny edge density	$\frac{1}{N}\sum_{p=1}^{N} C_p$, where $C_p \in 0,1$ denotes the Canny edge map
31	feature_030	Sharpness / edge / gradient	Tenengrad mean	$\frac{1}{N}\sum_{p=1}^{N}\left(G_{x,p}^2+G_{y,p}^2\right)$
32	feature_031	Sharpness / edge / gradient	Tenengrad standard deviation	$\mathrm{Std}\left(G_x^2+G_y^2\right)$
33	feature_032	Sharpness / edge / gradient	FFT high-frequency energy ratio	$\frac{\sum_{(u,v)\square\Omega_{\mathrm{h}}}\lvert \mathrm{F}(I)(u,v)\rvert^2}{\sum_{(u,v)\square\Omega}\lvert \mathrm{F}(I)(u,v)\rvert^2+\epsilon}$
34	feature_033	Sharpness / edge / gradient	Normalized gradient magnitude entropy	$-\sum_b \mathrm{h}_b^M \log\left(\mathrm{h}_b^M+\epsilon\right)$, where h_b^M is the normalized histogram of M
35	feature_034	Noise / local variation	Gaussian residual standard deviation	$\mathrm{Std}(R)$, where $R=I-\mathrm{G}_\sigma(I)$
36	feature_035	Noise / local variation	Mean absolute Gaussian residual	$\frac{1}{N}\sum_{p=1}^{N}\lvert R_p\rvert$
37	feature_036	Noise / local variation	95th percentile absolute residual	$P_{95}(\lvert R\rvert)$
38	feature_037	Noise / local variation	Local variance mean	$\frac{1}{N}\sum_{p=1}^{N} V_{\mathrm{loc}}(p)$, where $V_{\mathrm{loc}}(p)$ is the variance within a local window centered at p
39	feature_038	Noise / local variation	Local variance standard deviation	$\mathrm{Std}(V_{\mathrm{loc}})$
40	feature_039	Noise / local variation	Gray standard deviation / residual standard deviation ratio	$\frac{\sigma_I}{\mathrm{Std}(R)+\epsilon}$
41	feature_040	Texture / structure, GLCM	GLCM contrast	$\sum_{i,j}(i-j)^2 P(i,j)$
42	feature_041	Texture / structure, GLCM	GLCM dissimilarity	$\sum_{i,j}\lvert i-j\rvert P(i,j)$
43	feature_042	Texture / structure, GLCM	GLCM homogeneity	$\sum_{i,j}\frac{P(i,j)}{1+(i-j)^2}$
44	feature_043	Texture / structure, GLCM	GLCM energy	$\sqrt{\sum_{i,j} P(i,j)^2}$

No.	Feature ID	Feature group	Descriptor	Definition / formulation
45	feature_044	Texture / structure, GLCM	GLCM correlation	$\sum_{i,j} \frac{(i-\mu_i)(j-\mu_j) P(i,j)}{\sigma_i \sigma_j + \epsilon}$
46	feature_045	Texture / structure, GLCM	GLCM angular second moment	$\sum_{i,j} P(i,j)^2$
47	feature_046	Texture / structure, GLCM	GLCM entropy	$-\sum_{i,j} P(i,j) \log(P(i,j)+\epsilon)$
48	feature_047	Texture / structure, GLCM	GLCM maximum probability	$\max_{i,j} P(i,j)$
49	feature_048	Gabor directional texture	Gabor absolute response, 0°	$\frac{1}{N}\sum_{p=1}^{N} \lvert G_{0°,p} \rvert$
50	feature_049	Gabor directional texture	Gabor absolute response, 45°	$\frac{1}{N}\sum_{p=1}^{N} \lvert G_{45°,p} \rvert$
51	feature_050	Gabor directional texture	Gabor absolute response, 90°	$\frac{1}{N}\sum_{p=1}^{N} \lvert G_{90°,p} \rvert$
52	feature_051	Gabor directional texture	Gabor absolute response, 135°	$\frac{1}{N}\sum_{p=1}^{N} \lvert G_{135°,p} \rvert$
53	feature_052	Gabor directional texture	Standard deviation of four directional Gabor responses	Std($\bar{G}0°,\bar{G}45°,\bar{G}90°,\bar{G}135°$)), where $\bar{G}\theta = \frac{1}{N}\sum p=1^{N}$

Supplementary Note S1. Reliability-Aware Soft-Target and Sample-Weight Construction

Parameter-setting rationale. The constants in Equations (S2)–(S4) were fixed before the main cross-validation experiments and were held constant across all folds. They were selected to preserve the manually assigned class as the dominant soft-target component, restrict probability transfer primarily to adjacent annotatability levels, and prevent excessive smoothing under low annotation reliability or uncertain ROI localization. Across all admissible values of a_i and s_i, the probability assigned to the manual class remains above 0.70. No parameter was optimized using a held-out test fold.

Let $a_i \in [0,1]$ denote the fold-safe normalized Annotation Reliability Score (ARS) of sample i, and let $s_i \in [0,1]$ denote its ROI-localization uncertainty. For each cross-validation fold, the normalization mapping from the raw ARS to a_i is estimated using only the training split and is then applied unchanged to the corresponding validation and test splits.

For an accepted eye-region detection with confidence c_i,the localization uncertainty is defined as

$$s_i = \begin{cases} 0, & c_i \geq 0.50, \\ \frac{0.50-c_i}{0.50-0.25}, & 0.25 \leq c_i < 0.50, \\ 1, & \text{if no accepted ROI is available.} \end{cases} \quad \text{(S1)}$$

Thus, a high-confidence ROI receives no localization penalty, an accepted low-confidence ROI

receives a linearly increasing uncertainty value, and a full-image fallback receives the maximum uncertainty.

The manually assigned H/M/L hard label is retained as the fixed evaluation target. During neural-model training, it is converted into an asymmetric ordinal soft target

$$\tilde{y}_i=\left(\tilde{y}_{i,H},\tilde{y}_{i,M},\tilde{y}_{i,L}\right),\ \ \sum_{k\square\{H,M,L\}}\tilde{y}_{i,k}=1.$$

For a sample labeled as High,

$$\tilde{y}_{i,M}=min[0.08+0.18(1-a_i)+0.05s_i,0.25],$$
$$\tilde{y}_{i,L}=min(0.03s_i,0.05),$$
$$\tilde{y}_{i,H}=1-\tilde{y}_{i,M}-\tilde{y}_{i,L}. \qquad \text{(S2)}$$

High samples are therefore softened mainly toward the adjacent Medium level, while only a small probability is assigned to Low under uncertain localization.

For a sample labeled as Medium, two directional terms are defined as

$$u_i=a_i(1-s_i), \qquad d_i=\min[1,s_i+0.5(1-a_i)].$$

The corresponding soft target is

$$\tilde{y}_{i,H}=\min(0.06+0.14u_i,0.22),$$
$$\tilde{y}_{i,L}=\min(0.06+0.16d_i,0.25), \qquad \text{(S3)}$$
$$\tilde{y}_{i,M}=1-\tilde{y}_{i,H}-\tilde{y}_{i,L}.$$

This formulation allows reliable Medium samples to retain a limited probability toward High, while low annotation reliability or uncertain ROI localization increases the probability assigned to Low.

For a sample labeled as Low,

$$\tilde{y}_{i,H}=0,$$
$$\tilde{y}_{i,M}=\min[0.03+0.07(1-s_i)(1-a_i),0.10], \qquad \text{(S4)}$$
$$\tilde{y}_{i,L}=1-\tilde{y}_{i,M}.$$

Low samples are softened only toward the adjacent Medium level, and no probability is assigned directly to High. After construction, all soft-target components are clipped to [0,1], normalized to sum to one, and checked to ensure that the manually assigned class remains the dominant component.

A reliability-aware sample weight is further computed as

$$\rho_i=0.70a_i+0.30(1-0.5s_i),$$
$$w_i=\text{clip}\left(0.70+0.30\rho_i,0.70,1.00\right), \qquad \text{(S5)}$$

with $w_i \geq 0.85$ enforced for Low samples. This weighting reduces the influence of samples with weak geometric consistency or uncertain ROI localization without removing them from training or changing their hard labels.

The soft targets $\tilde{y}_i$ and sample weights w_i are used only during the training of neural single-branch predictors and trainable fusion baselines. LateAvg itself introduces no additional joint-training objective, and the reliability variables do not participate in posterior averaging during inference. Scalar-IQA and conventional machine-learning baselines use the fixed hard labels without reliability-aware weighting. All validation and test metrics are computed strictly against the original manual_label.

Supplementary Table S5. Sensitivity of LateAvg to fixed posterior fusion weights.

For sensitivity analysis, the fused posterior was expressed as $p_i(\alpha)=\alpha p_i^H+(1-\alpha)p_i^D$, where α denotes the HQD posterior weight. The branch classifiers were not retrained, and the analysis was conducted only to evaluate the robustness of the predefined equal-weight configuration. All results are reported as the mean ± sample standard deviation across the five held-out test folds.

**HQD weight (α)**	**DQE weight (1-α)**	**Macro-F1**	**H-F1**	**M-F1**	**L-F1**
0.0	1.0	0.8385 ± 0.0516	0.9595 ± 0.0206	0.7403 ± 0.1105	0.8156 ± 0.0641
0.1	0.9	0.8411 ± 0.0518	0.9617 ± 0.0216	0.7455 ± 0.1125	0.8161 ± 0.0649
0.2	0.8	0.8434 ± 0.0550	0.9634 ± 0.0243	0.7496 ± 0.1150	0.8172 ± 0.0685
0.3	0.7	**0.8528 ± 0.0582**	**0.9661 ± 0.0304**	0.7676 ± 0.1161	0.8247 ± 0.0706
0.4	0.6	0.8519 ± 0.0633	0.9601 ± 0.0340	0.7672 ± 0.1246	0.8283 ± 0.0744
0.5	0.5	0.8510 ± 0.0612	0.9549 ± 0.0357	0.7685 ± 0.1181	**0.8296 ± 0.0757**
0.6	0.4	0.8517 ± 0.0572	0.9552 ± 0.0383	0.7719 ± 0.1118	0.8280 ± 0.0772
0.7	0.3	0.8489 ± 0.0654	0.9493 ± 0.0446	**0.7689 ± 0.1246**	0.8285 ± 0.0809
0.8	0.2	0.8347 ± 0.0748	0.9324 ± 0.0600	0.7516 ± 0.1356	0.8202 ± 0.0892
0.9	0.1	0.8313 ± 0.0757	0.9299 ± 0.0634	0.7495 ± 0.1321	0.8145 ± 0.0944
1.0	0.0	0.8232 ± 0.0800	0.9208 ± 0.0653	0.7465 ± 0.1269	0.8022 ± 0.1174

Note: *α=0 and α=1 correspond to DQE-only and HQD-only, respectively. The predefined LateAvg configuration uses α=0.5. The weight grid was used only for robustness analysis and was not used to redefine the final model.*

Supplementary Table S5 shows that LateAvg remains stable across a broad range of fixed posterior weights. Although the highest diagnostic mean Macro-F1 of 0.8528 is observed at α=0.3, it exceeds the predefined equal-weight result by only 0.0018. All settings from α=0.3 to 0.7 remain within 0.005 Macro-F1 of the grid maximum, and the variation among α=0.4, 0.5, and 0.6 is below 0.001. These results support equal posterior averaging as a stable operating point rather than a narrowly tuned configuration.

Supplementary Table S6. Fold-wise Macro-F1 scores of LateAvg and strong representation, fusion, and fine-tuning baselines.

This table reports fold-level Macro-F1 scores for LateAvg and the strong representation, fusion, and fine-tuning baselines to assess performance consistency across the five held-out test folds.

**Method**	**Fold 0 (n = 327)**	**Fold 1 (n = 282)**	**Fold 2 (n = 343)**	**Fold 3 (n = 262)**	**Fold 4 (n = 286)**	**Mean**	**Std**
HQD-only	0.8287	0.8870	0.7508	0.9149	0.7345	0.8232	**0.0800**
DQE-only	0.8080	0.8738	0.8234	0.9077	0.7795	0.8385	0.0516
GMU-Fusion	0.8151	0.8355	**0.8770**	0.9165	**0.7870**	0.8462	0.0512
MANIQA-LateStage-FT	**0.9014**	0.8698	0.7884	0.8872	0.7766	0.8447	0.0580
LateAvg (Ours)	0.8189	**0.8854**	0.8394	**0.9350**	0.7761	**0.8510**	0.0612

Note: *Mean and Std denote the arithmetic mean and sample standard deviation across the five folds, respectively, with the standard deviation calculated using ddof=1. All methods were evaluated on identical held-out folds under the five-fold cross-validation protocol.*

LateAvg achieves the highest mean Macro-F1 among these strong alternatives, although the

fold-wise ranking is not uniform. It outperforms DQE-only on four of the five folds and exceeds HQD-only, GMU-Fusion, and MANIQA-LateStage-FT on three folds each. The results therefore support a higher aggregate performance for LateAvg while remaining consistent with the absence of statistically significant differences from these strong baselines under the five-fold paired tests.

Supplementary Table S7. Hyperparameter configurations of competing methods.

Method	Input / Feature	Main Hyperparameters	Training / Selection Strategy
Random Forest	53D HQD	n_estimators=300; max_depth={None,10}; min_samples_leaf=5; max_features={sqrt,log2}	Grid search within each fold
XGBoost	53D HQD	n_estimators=200; max_depth=3; learning_rate={0.03,0.1} subsample={0.8,1.0}; colsample_bytree=0.8; reg_lambda={1,5}	Fold-wise validation Macro-F1
Logistic Regression	53D HQD	penalty=l2; solver=lbfgs; C={0.01,0.1,1}	Fold-wise validation
SVM	53D HQD	kernel=RBF; C={1,10,100}; gamma={scale,0.01}	Fold-wise validation
GMU-Fusion	HQD+DQE	hidden_dim=128; fusion_dim=128; dropout=0.2; sigmoid feature gate	AdamW; lr=1e-4; weight decay=1e-4; batch=64; epochs=50; patience=10
MANIQA-LateStage-FT	MANIQA feature	classifier 384-128-3; dropout=0.3; partial backbone fine-tuning	AdamW; backbone lr=5e-6; classifier lr=1e-4; weight decay=1e-4; batch=16; epochs=20; patience=6
